# Physiological Lipid Palmitoylcarnitine Constrains Factor Xa Signalling Through PAR-2 to Attenuate ERK-Driven Inflammation and Preserve Mitochondrial Membrane Potential in Chondrocytes

**DOI:** 10.3390/biomedicines14081680

**Published:** 2026-07-27

**Authors:** Rajashree Patnaik, Yajnavalka Banerjee

**Affiliations:** College of Medicine, Mohammed Bin Rashid University of Medicine and Health Sciences, Dubai Health, Dubai 505055, United Arab Emirates; rajashree.patnaik@dubaihealth.ae

**Keywords:** palmitoylcarnitine, factor Xa, protease-activated receptor-2 (PAR-2), ERK/MAPK signalling, acylcarnitine–protease interaction, coagulation protease signalling, chondrocytes

## Abstract

**Background:** Factor Xa (FXa) is increasingly recognized as a non-hemostatic signaling protease that amplifies inflammatory responses through protease-activated receptor-2 (PAR-2), but its contribution to osteoarthritis-relevant chondrocyte signaling remains incompletely defined. A central unresolved question is whether an endogenous ligand of FXa can selectively dampen its receptor-mediated signaling output. **Objectives:** We investigated whether palmitoylcarnitine (PalCar), an endogenous long-chain acylcarnitine previously shown to bind FXa, modulates FXa-driven inflammatory and catabolic responses in bone marrow mesenchymal stem cell (BMSC)-derived chondrocytes, and the extent to which that output is hard-wired through PAR-2. **Methods:** BMSC-derived chondrocytes, validated histologically and by COL2A1 expression, were challenged with FXa (1 U/mL, 24 h) alone or together with PalCar (0.5 and 1.0 µg/mL). Cytokine, catabolic and signaling responses were assessed by RT-qPCR, Western blotting and ELISA; surface RANK and RANKL by flow cytometry; and mitochondrial membrane potential by Rhodamine 123. Receptor dependence was tested by siRNA-mediated PAR-2 silencing, and data were analyzed by one-way ANOVA with Dunnett’s post hoc test. **Results:** FXa stimulation induced PAR-2 expression, increased TNF-α, IL-1β, and MCP-1 production, enhanced ERK1/2 activation, upregulated the catabolic mediators SOX4 and ADAMTS5, promoted surface RANK/RANKL expression, and impaired mitochondrial membrane potential. PalCar at 0.5 and 1.0 µg/mL significantly attenuated these responses at both transcript and protein levels and restored mitochondrial membrane potential under FXa-induced stress. Mechanistically, siRNA-mediated PAR-2 silencing substantially blunted, but did not completely quench, the inflammatory response to FXa, identifying PAR-2 as the dominant, though not exclusive, signaling conduit in this system. **Conclusions:** By combining pharmacological challenge with receptor knockdown, our data define PalCar as an endogenous, PAR-2-directed suppressor of the FXa–PAR-2–ERK inflammatory axis in chondrocytes. Given emerging evidence linking osteoarthritis with increased cerebrovascular risk, PalCar-sensitive FXa signaling may represent a mechanistically relevant intersection between joint inflammation and broader thrombo-inflammatory disease biology, providing a mechanistic basis for evaluating FXa-responsive lipid pathways in osteoarthritis and related thrombo-inflammatory states.

## 1. Introduction

Osteoarthritis (OA) is increasingly understood as a disease driven not solely by mechanical cartilage degradation but also by persistent low-grade inflammatory signaling within the joint microenvironment [1,2]. Chondrocytes, synovial fibroblasts, and subchondral bone cells produce and respond to proinflammatory cytokines including tumour necrosis factor-α (TNF-α), interleukin-1β (IL-1β), and monocyte chemoattractant protein-1 (MCP-1), which collectively activate catabolic cascades involving matrix metalloproteinases and aggrecanases. These mediators disrupt the anabolic-catabolic equilibrium essential for cartilage homeostasis, accelerating extracellular matrix loss [3]. Despite recognition of the inflammatory component, the upstream protease signaling events that initiate and sustain cytokine production in chondrocytes remain incompletely characterized.

Contemporary frameworks increasingly conceptualise OA as a disease of the whole joint as an organ, in which articular cartilage, synovium, subchondral bone, and the infrapatellar fat pad sustain a self-perpetuating inflammatory circuit rather than a purely mechanical wear phenomenon [4]. The infrapatellar fat pad is itself an active osteoarthritic tissue rather than a passive space filler, secreting pro-inflammatory cytokines and adipokines, among them interleukin-6, interleukin-8 and TNF-α, that act upon the adjacent cartilage and synovium [5]; moreover, its anatomical, vascular and functional continuity with the synovial membrane is now recognised to constitute a single anatomo-functional unit, such that inflammatory signalling within the fat pad and the synovium is effectively shared [6]. Articular cartilage is an avascular, aneural tissue whose load-bearing competence depends on a highly ordered extracellular matrix dominated by type II collagen, aggrecan-bound proteoglycans, and interstitial water, and its structural and viscoelastic properties are progressively compromised as the disease advances [7,8]. Within this matrix the chondrocyte is the sole resident cell and therefore the principal integrator of catabolic and inflammatory cues; under sustained stress it loses its stable phenotype, acquires a senescence-associated secretory programme, and amplifies the release of cytokines and matrix-degrading enzymes that act back on neighbouring cells [4]. This chronic, low-grade inflammatory state, frequently termed inflammaging, is now recognised to interlock with cellular senescence, oxidative stress, and disturbances in trace-element and micronutrient homeostasis that together erode cartilage anabolism [4,9]. Mitochondrial dysfunction has emerged as a central node in this circuit, since dissipation of the mitochondrial membrane potential and the attendant accumulation of reactive oxygen species not only impair chondrocyte bioenergetics but also potentiate NF-κB- and MAPK-dependent inflammatory signalling, rendering bioenergetic failure both a consequence and an amplifier of the catabolic programme [10]. Many of these converging inputs are ultimately transduced through the mitogen-activated protein kinase cascade, and ERK1/2 in particular has been identified as a dominant, spatially restricted node of MAPK activation in osteoarthritic joint tissue and as a tractable therapeutic target [11,12]. Taken together, these advances refocus the search for OA-initiating events onto the proximal receptors and proteases that ignite MAPK/ERK-driven inflammation, and they furnish the conceptual rationale for interrogating a coagulation-protease input to this cascade.

Coagulation proteases have emerged as important non-hemostatic signaling molecules. Factor Xa (FXa), a serine protease central to the coagulation cascade, activates protease-activated receptors (PARs), a family of G protein-coupled receptors cleaved by extracellular proteases [13]. Among these, PAR-2 is of particular interest in inflammatory biology. FXa cleaves the extracellular N-terminal domain of PAR-2 to generate a tethered ligand that triggers intracellular signalling cascades including mitogen-activated protein kinase (MAPK) pathways, NF-κB activation, and cytokine transcription [14,15]. PAR-2 activation has been implicated in inflammatory arthritis, synovial inflammation, and cartilage catabolism, positioning the FXa–PAR-2 axis as a plausible contributor to OA pathobiology [16,17]. Consistent with a causal contribution, PAR-2 is over-expressed in human osteoarthritic cartilage and in cytokine-stimulated chondrocytes, and pharmacological PAR-2 antagonism attenuates inflammatory, catabolic, and senescence-associated responses while preserving matrix integrity in preclinical OA models [18]. Beyond the joint, inhibition of FXa suppresses PAR-2-dependent ERK1/2 activation and its downstream inflammatory and apoptotic sequelae in other tissues, directly implicating the FXa–PAR-2–ERK module as a druggable inflammatory axis [19]. More broadly, the recognition that coagulation proteases act as immune effectors underpins the concept of thrombo-inflammation, a bidirectional coupling of haemostatic and inflammatory pathways that is increasingly implicated in chronic degenerative and cardiovascular disease and that provides a mechanistic frame for linking joint-localised FXa signalling to systemic inflammatory risk [20]. Our laboratory has previously established an in vitro inflammatory chondrocyte model using BMSC-derived chondrocytes stimulated with lipopolysaccharide and characterized cytokine production and catabolic marker expression in that system [21,22]. The present study adapts this validated chondrocyte platform to interrogate FXa as a protease-specific inflammatory stimulus, providing a tractable system for dissecting coagulation protease-driven signalling in chondrocyte biology.

Palmitoylcarnitine (16:0-acylcarnitine; PalCar) is an endogenous long-chain acylcarnitine generated during mitochondrial fatty acid β-oxidation. It has been previously demonstrated, using untargeted and targeted metabolomics coupled with surface plasmon resonance, that PalCar and related long-chain acylcarnitines bind directly to FXa outside its γ-carboxyglutamic acid (Gla) domain and inhibit prothrombinase activity, identifying acylcarnitines as a novel class of endogenous anticoagulant lipids [23]. That study also showed that low plasma acylcarnitine levels are associated with increased venous thromboembolism risk. These observations raise the question of whether PalCar, through its interaction with FXa, can modulate not only hemostatic but also non-hemostatic FXa signaling, specifically the inflammatory output mediated through PAR-2.

Whether PalCar can attenuate FXa-driven inflammatory and catabolic signaling in chondrocytes has not been examined. It remains unknown whether such modulation would involve suppression of PAR-2 expression, dampening of downstream MAPK signaling, reduction in catabolic effector pathways, or preservation of mitochondrial function under inflammatory stress.

The objective of the present study was to determine whether PalCar attenuates FXa-induced inflammatory signalling in BMSC-derived chondrocytes by modulating the PAR-2–ERK signaling axis, suppressing catabolic mediator expression, and preserving mitochondrial membrane potential. We further employed siRNA-mediated PAR-2 knockdown to assess the degree to which the FXa-induced inflammatory program depends on PAR-2 as a proximal signaling receptor. Conceptually, this design moves beyond a description of which markers change and instead asks a defined mechanistic question: does an endogenous FXa-binding lipid restrain protease-driven signalling, and is that restraint routed predominantly through a single receptor? Coagulation proteases such as FXa are now recognized to act through protease-activated receptors and tissue factor to reprogramme gene expression in non-haemostatic settings [4,23], yet whether this output can be tuned by a physiological lipid ligand of the protease itself has not been tested in cartilage-lineage cells.

## 2. Materials and Methods

### 2.1. Ethics Statement

This study employed exclusively in vitro experiments using commercially acquired cell lines and did not involve human subjects, animal models, or patient-derived samples. The study was exempt from formal ethical review as determined by the Institutional Review Board (IRB) of Mohammed Bin Rashid University of Medicine and Health Sciences (MBRU), Dubai, United Arab Emirates.

### 2.2. Cell Culture

Human bone marrow mesenchymal stem cells (BMSCs) were obtained from AddexBio Technologies (San Diego, CA, USA). As this is a characterised, commercially available BMSC line rather than a primary isolate from recruited patients, the study did not involve multiple independent donors; all experiments were performed with a single commercial cell line (catalogue number, P0004011: AddexBio, San Diego, CA, USA; lot number: 0002237) at passage 5. Accordingly, a biological replicate was defined as an independent chondrogenic differentiation performed on a separate occasion from an independently expanded culture of this line, with three independent biological replicates (*n* = 3) analysed per condition unless otherwise stated, each assayed in technical triplicate. Cells were cultured in mesenchymal stem cell growth medium (MSCM; AddexBio Technologies, San Diego, CA, USA) supplemented with 10% fetal bovine serum (FBS; HiMedia, Thane, Maharashtra, India) and 1% penicillin-streptomycin (HiMedia, Thane, Maharashtra, India) at 37 °C in a humidified atmosphere containing 5% CO_2_. Medium was replaced every three days. Cells were passaged at 90% confluence using trypsin/EDTA (HiMedia, Thane, Maharashtra, India).

### 2.3. Chondrogenic Differentiation

Chondrogenic differentiation was performed as previously standardized [9]. Briefly, BMSCs at passage 5 were counted using an automated cell counter (CellDrop; DeNovix, Wilmington, NC, USA), and 2 × 10^6^ cells were pelleted by centrifugation at 1000 rpm for 10 min in 15 mL polypropylene tubes. Pellets were resuspended in 2 mL complete MesenCult™-ACF chondrogenic differentiation medium (STEMCELL™ Technologies, Vancouver, BC, Canada; Catalogue Number, #05456), distributed into four tubes (0.5 mL each), and centrifuged at 1000 rpm for 10 min at 25 °C. Pellets were incubated at 37 °C with 5% CO_2_ for 21 days, with medium renewal every three days. Pellets were gently agitated during medium changes to prevent adhesion. For cryopreservation, differentiated chondrocytes were pelleted, resuspended in freezing medium (DMEM with 5% DMSO), stored overnight at −80 °C, and transferred to liquid nitrogen.

### 2.4. Histological Staining

Cell pellets were fixed in 10% neutral-buffered formalin for 24 h, dehydrated through graded ethanol, cleared in xylene, and embedded in paraffin. Sections (6 µm) were cut using a microtome (Leica RM2255, Heidelberg, Germany). Chondrogenic phenotype was verified by collagen type II immunostaining, together with Alcian Blue and Toluidine Blue staining (Abcam, Waltham, MA, USA), according to established protocols [9]. Sections were examined using an Olympus BX63 microscope equipped with an Olympus DP80 camera and CellSens Dimension 2.3 software at 20× and 40× magnification.

### 2.5. Cell Viability Assay

Cytotoxicity was assessed using the MTT (3-(4,5-dimethylthiazol-2-yl)-2,5-diphenyltetrazolium bromide) assay as previously described [9]. MTT (Catalogue Number, HY-15924) was purchased from Med Chem Express, Monmouth Junctio, NJ, USA. Differentiated chondrocytes were treated with FXa (0, 0.5, 1.0, 2.0, 5.0 µg/mL) or PalCar (0, 0.1, 0.25, 0.5, 1.0 µg/mL) for 24 h. Following 24 h treatment, cells were incubated with MTT (5 mg/mL; 20 µL/well) for 4 h at 37 °C. Formazan crystals were dissolved in DMSO, and absorbance was measured at 570 nm using a Hidex microplate reader (Turku, Finland). Cell viability was expressed as % of control: (100 × (ODsample − ODblank)/ODcontrol), with MTT in DMSO as blank.

### 2.6. FXa Stimulation and PalCar Treatment

Differentiated chondrocytes were stimulated with FXa (Molecular Depot, San Diego, CA, USA; Catalogue Number, B2010471) at 1 unit per well in 6-well plates for 24 h at 37 °C with 5% CO_2_ to generate the inflammatory chondrocyte model. FXa has been shown to induce proinflammatory cytokine expression, including TNF-α, through PAR-2-dependent ERK1/2 signaling in multiple cell types. Exploiting this established signaling axis, the proinflammatory status of the FXa-stimulated chondrocyte model was validated by quantification of TNF-α secretion using ELISA, as described below. Only cultures demonstrating significant TNF-α elevation relative to unstimulated controls were used for subsequent PalCar treatment experiments. Following confirmation of inflammatory activation, cells were treated with PalCar (Larodan AB, Solna, Sweden; Catalogue Number, 17-1600-7) at 0, 0.1, 0.25, 0.5, or 1.0 µg/mL for an additional 24 h. DMSO in complete culture medium served as the vehicle control, with the final concentration maintained at 0.1% (*v*/*v*).

### 2.7. Enzyme-Linked Immunosorbent Assay (ELISA)

Conditioned medium was collected from chondrocyte cultures after treatment and centrifuged at 5000 rpm for 10 min at 4 °C. TNF-α, IL-1β, and MCP-1 concentrations were quantified using commercial ELISA kits (Abcam, Boston, MA, USA) (Catalogue Numbers: TNF-α, ab181421; IL-1β, ab181421 and MCP-1, ab179886) according to the manufacturer’s instructions. Absorbance was measured at 450 nm with 620 nm reference using a Hidex microplate reader. Experiments were performed in triplicate.

### 2.8. RT-qPCR

Total RNA was extracted from control and treated chondrocytes (Catalogue number, MB13402; NZYtech, Lisboa, Portugal). First-strand cDNA was synthesized using the First-Strand cDNA Synthesis Kit (Catalogue number, NP100042; OriGene, Herford, Germany). Real-time quantitative PCR was performed using SYBR Green chemistry on a QuantStudio 5 Flex instrument (Applied Biosystems, San Diego, CA, USA). GAPDH served as the endogenous reference gene, and relative expression was calculated using the 2^−ΔΔCt^ method. Primer sequences employed for SYBR Green-based amplification are provided in Supplementary Table S1. The annealing temperature for all primer sets was set at 60 °C. PCR amplification was performed with an initial denaturation at 95 °C for 3 min, followed by 40 cycles of denaturation at 95 °C for 10 s and annealing/extension at 60 °C for 30 s.

### 2.9. Western Blotting

Total protein was extracted using lysis buffer containing 0.5% SDS (Invitrogen, Waltham, MA, USA) supplemented with protease inhibitors (Catalogue Number, 78430; Thermofisher Scientific, San Diego, CA, USA). Protein concentrations were determined by BCA assay (Catalogue Number, A65453; Thermofisher Scientific, San Diego, CA, USA). Equal amounts (15 µg) of protein were resolved on 10% SDS-PAGE gels and transferred to nitrocellulose membranes (Bio-Rad Laboratories, Vancouver, BC, Canada). Membranes were blocked with 5% BSA in TBS (Pierce, San Diego, CA, USA) for 60 min at 4 °C, washed with TBS-Tween 20 (TTBS), and incubated overnight at 4 °C with primary antibodies against mouse anti-human PAR-2 (catalogue number, ab184673; mouse monoclonal; clone number: SAM11; Abcam), rabbit anti-human ERK1/2 (Catalogue number, ab184699; rabbit monoclonal: clone number, EPR17526; Abcam, San Diego, CA, USA), rabbit anti-human phospho-ERK1/2 (Thr202/Tyr204) (catalogue number, 9101S; rabbit polyclonal; Cell Signalling, Boston, MA, USA), rabbit anti-human SOX4 (catalogue number, ab316850; rabbit monoclonal: clone number, EPR28132-51; Abcam, San Diego, CA, USA), rabbit anti-human COL2A1 (catalogue number, ab307674; rabbit polyclonal; Abcam, San Diego, CA, USA), and rabbit anti-human ADAMTS-5 [catalogue number, ab41037; rabbit polyclonal; Abcam] (1:1000) in SuperBlock (1:10 in TTBS). After washing, membranes were incubated with HRP-conjugated anti-mouse [catalogue number, ab205719; polyclonal; Abcam, San Diego, CA, USA] or anti-rabbit secondary antibodies (1:2000; Abcam) (catalogue number, ab6728; polyclonal; Abcam, San Diego, CA, USA) for 1 h at room temperature. Chemiluminescence was detected using SuperSignal ULTRA (Pierce) and imaged on Kodak BioMax film (GE Healthcare, Vancouver, BC, Canada). Densitometric analysis was performed using ImageJ software (Fiji distribution of ImageJ2, version 1.54f, National Institutes of Health, Bethesda, MD, USA) with GAPDH (Catalogue number, ab9482; mouse monoclonal: clone number, mAbcam 9484; Abcam) as loading control. For densitometric quantification, the integrated intensity of each target band was measured in ImageJ and normalised to that of the GAPDH loading control resolved from the same lane and membrane; the normalised values were then expressed relative to the designated reference condition and reported as fold-change, with the densitometric values from each of the three independent biological replicates displayed as individual data points on the corresponding bar graphs. Molecular-weight positions, assigned against a co-resolved protein ladder, are indicated on all blots, and uncropped, full-length images of every immunoblot, together with their corresponding loading-control membranes, are provided in the Supplementary Information. All antibodies used in this study were validated by the respective manufacturers for specificity and performance in the relevant applications. All experiments were performed in triplicate.

### 2.10. Flow Cytometry

For surface RANK and RANKL analysis, chondrocytes were trypsinized, centrifuged at 1800 rpm for 5 min at 4 °C, and resuspended in PBS at 1 × 10^6^ cells/mL. For RANK detection, cells were incubated with mouse anti-human RANK-PE (20 µg/mL; Abcam) (Catalogue number, ab233817; mouse monoclonal: clone number, 8H8B2; Abcam) for 30 min at 4 °C. For RANKL, cells were incubated with unconjugated rabbit anti-human RANKL (15 µg/mL; Abcam) (catalogue number, ab216484; rabbit polyclonal; Abcam) followed by FITC-conjugated goat anti-rabbit secondary antibody (7.5 µg/mL; Abcam) (catalogue number, ab97050; polyclonal; Abcam) for 30 min at 4 °C. Cells were analyzed on a FACS Calibur flow cytometer (BD Biosciences, Vancouver, BC, Canada). A total of 20,000 events were acquired per sample. The gating strategy involved initial exclusion of debris based on forward scatter (FSC) and side scatter (SSC) properties, followed by doublet discrimination using FSC-height versus FSC-area parameters. RANK-PE- or RANKL-FITC-positive cells were then identified within the gated singlet population using the corresponding isotype controls to define background fluorescence. In addition, unstained and single-stained controls were prepared in parallel for each fluorophore; together with the matched isotype controls, these were used to position the boundary of the positive gate and to correct for background and spectral spillover, in accordance with established criteria for the determination of positivity. For every sample, both the median fluorescence intensity (MFI) of the RANK-PE or RANKL-FITC signal and the percentage of positive cells, defined relative to the unstained and isotype thresholds, were recorded. Representative plots of the complete gating strategy, FSC/SSC debris exclusion, FSC-height versus FSC-area doublet discrimination, and positive-gate definition against the unstained and isotype controls are provided in Supplementary Figure S2. Data were analyzed using FlowJo software (version 10.8.1; FlowJo, Ashland, OR, USA).

### 2.11. Mitochondrial Membrane Potential Assessment

Mitochondrial membrane potential (ΔΨm) was assessed using Rhodamine 123 (Thermo Fisher, CA, USA). Chondrocytes from the inflammatory model treated with 0.25 or 1.0 µg/mL PalCar were washed three times with PBS and stained with 2 µM Rhodamine 123 for 15 min at room temperature. After washing to remove unbound dye, fluorescent images were captured on an Olympus fluorescence microscope (Olympus, Tokyo, Japan). Fluorescence intensity was quantified using ImageJ software (version 1.54t), and ΔΨm dissipation was expressed as percentage reduction in green fluorescence intensity relative to controls.

### 2.12. siRNA-Mediated PAR-2 Knockdown

PAR-2 expression was silenced using small interfering RNA (siRNA) targeting human PAR-2/F2RL1 (SR30002; OriGene Technologies, Herford, Germany). Cells were transfected with siRNA using siTran 2.0 siRNA transfection reagent (TT320001, OriGene Technologies, Herford, Germany) according to the manufacturer’s optimized protocol. Briefly, cells were seeded at a density of 2 × 10^5^ cells/well in six-well plates and allowed to reach approximately 70% confluence prior to transfection. siRNA was used at a final concentration of 10 nM. siRNA–transfection reagent complexes were prepared in serum-free medium and incubated at room temperature for 15 min before being added to the cells. Cells were then incubated at 37 °C in a humidified 5% CO_2_ atmosphere for 48 h to allow gene silencing. Knockdown efficiency was confirmed by Western blot analysis of PAR-2 protein expression. Following silencing, cells were stimulated with FXa (1 unit) for 24 h in the presence or absence of palmitoylcarnitine (0.5 and 1.0 µg/mL), and subsequently assessed for PAR-2 protein expression and inflammatory readouts, including TNF-α.

### 2.13. Statistical Analysis

Data are expressed as mean ± SD of *n* = 3 independent biological experiments, each representing a separately established, differentiated, treated, and analyzed BMSC-derived chondrocyte culture. Between-group differences were assessed by one-way analysis of variance (ANOVA) followed by Dunnett’s multiple-comparisons test, with the FXa-stimulated group specified as the reference (control) column against which the untreated, FXa + PalCar 0.5 µg/mL and FXa + PalCar 1.0 µg/mL groups were each compared; the resulting multiplicity-adjusted *p*-values are reported, and the individual data points for each biological replicate are displayed on the corresponding graphs. A *p*-value < 0.05 was considered significant. Analyses were conducted using GraphPad Prism v9.5.1 (GraphPad Software, San Diego, CA, USA).

## 3. Results

### 3.1. Characterization of BMSC-Derived Chondrocytes

**Figure 1:**
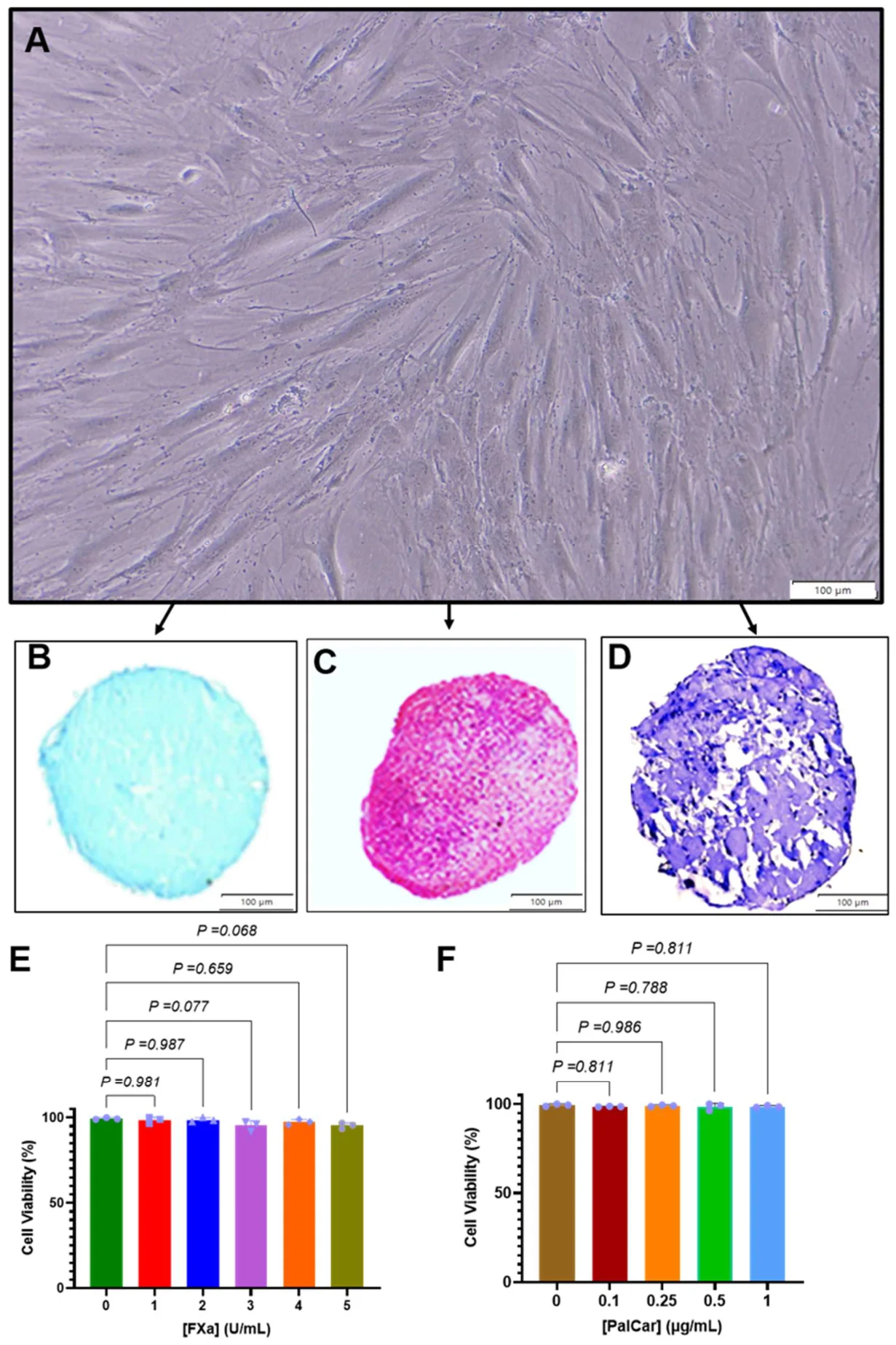
Characterization of BMSC-derived chondrocytes and assessment of FXa and PalCar cytotoxicity. (**A**) Representative phase-contrast image of human BMSCs showing spindle-shaped fibroblastic morphology at confluence. Chondrogenic differentiation after 21 days of pellet culture was confirmed by (**B**) Alcian blue staining, (**C**) collagen type II immunostaining, and (**D**) toluidine blue staining. (**E**,**F**) MTT assays showing that FXa (0–5.0 U/mL) and PalCar (0.1–1.0 µg/mL) did not significantly affect chondrocyte viability. Data are presented as mean ± SD from *n* = 3 independent biological experiments, with the individual data points for each replicate shown. Statistical comparisons were made by one-way ANOVA followed by Dunnett’s multiple-comparisons test against the untreated (0 U/mL FXa or 0 µg/mL PalCar) control column, and the exact multiplicity-adjusted *p*-values are shown. Scale bars: (**A**) 50 µm; (**B**–**D**) 100 µm.

Human BMSCs cultured in mesenchymal stem cell growth medium exhibited characteristic spindle-shaped fibroblastic morphology at confluence (Figure 1A). Following 21-day pellet culture in chondrogenic differentiation medium, histological assessment confirmed successful chondrocyte differentiation. Alcian blue staining demonstrated abundant sulfated glycosaminoglycan (GAG) deposition throughout the pellet matrix (Figure 1B). Collagen type II immunostaining confirmed the presence of the principal cartilage-specific collagen isoform within the differentiated pellet matrix (Figure 1C), consistent with commitment to a chondrogenic lineage. Toluidine blue staining exhibited metachromatic staining indicative of cartilaginous proteoglycan deposition (Figure 1D). The concordant detection of sulfated GAGs, type II collagen, and metachromatic proteoglycan accumulation validated the chondrogenic phenotype of the differentiated cells used in all subsequent experiments.

To complement this histological validation with a quantitative, phenotype-defining readout, we assessed COL2A1 (type II collagen), the principal cartilage-specific collagen and the canonical structural marker of the hyaline chondrocyte phenotype among the structural-gene and transcription-factor panel by which chondrocyte differentiation state is conventionally defined [24]. Western blotting and RT-qPCR demonstrated a marked increase in COL2A1 at both protein and transcript levels in differentiated chondrocytes relative to undifferentiated BMSCs, confirming acquisition of the chondrogenic phenotype (Supplementary Figure S1). The number of donors, cell passage, and definition of biological replicates are specified in the Materials and Methods.

### 3.2. FXa and PalCar Do Not Compromise Chondrocyte Viability

The FXa concentration used in this study, 1 U/mL (corresponding to 5.9 µg/mL), was selected based on preliminary MTT assays showing no significant reduction in chondrocyte viability across the tested range of 0.5–5.0 U/mL (Figure 1E). PalCar at 0.1–1.0 µg/mL was likewise non-cytotoxic (Figure 1F). These findings confirm that the observed downstream effects were not attributable to cytotoxicity and support the use of 1 U/mL FXa as a biologically effective in vitro inflammatory stimulus. Although this concentration is comparable in magnitude to reported plasma factor X levels [25], free active FXa in vivo is generated transiently and is tightly regulated [26]. Accordingly, 1 U/mL FXa should be regarded as an experimental challenge dose rather than a direct physiological equivalent. More precisely, 1 U/mL FXa (≈5.9 µg/mL) was applied as a continuously unopposed stimulus for 24 h, in the absence of the tissue factor pathway inhibitor and antithrombin that restrict FXa in vivo, and therefore constitutes a deliberately supraphysiological, mechanistic proof-of-concept challenge chosen to drive robust and reproducible pathway activation rather than an estimate of the local FXa concentration within the joint, notwithstanding that the osteoarthritic joint is itself a site of tissue-factor-driven coagulation activation and FXa generation [27]. PalCar, in contrast, was examined across 0.1–1.0 µg/mL (≈0.25–2.5 µM), a range within the low-micromolar concentrations characteristic of circulating long-chain acylcarnitines and consistent with the identity of palmitoylcarnitine as an endogenous FXa-binding anticoagulant lipid that acts outside the γ-carboxyglutamic acid domain [23]; the modulator was thus interrogated at physiologically grounded, non-cytotoxic concentrations, in deliberate contrast to the supraphysiological protease challenge.

### 3.3. Establishment of the FXa-Based Inflammatory Chondrocyte Model

To generate a validated proinflammatory chondrocyte system, differentiated BMSC-derived chondrocytes were stimulated with FXa at 1 U/mL for 24 h and inflammatory activation was confirmed by quantification of TNF-α secretion using ELISA. FXa-stimulated cultures exhibited a 58.13% increase in TNF-α secretion relative to unstimulated controls (** *p* < 0.01), confirming robust inflammatory activation. Model responsiveness was additionally corroborated at the level of the chondrogenic phenotype: FXa stimulation significantly reduced COL2A1 expression in differentiated chondrocytes at both protein and transcript levels (Supplementary Figure S1), consistent with a catabolic effect on cartilage-specific matrix synthesis, and, given that loss of COL2A1 is itself sufficient to accelerate chondrocyte hypertrophy and to promote osteoarthritis progression [28], furnishing a disease-relevant, phenotype-anchored index of FXa activity that complements the TNF-α readout. Concurrent Western blot analysis demonstrated that FXa stimulation markedly upregulated PAR-2 protein expression relative to unstimulated chondrocytes (refer below), consistent with prior reports that FXa induces PAR-2 mRNA and protein expression through a thrombin-independent mechanism involving NADPH oxidase-derived reactive oxygen species and NF-κB signaling in vascular smooth muscle cells [14]. These findings collectively confirm that FXa elicits both receptor upregulation and cytokine secretion in the chondrocyte system, consistent with the established capacity of FXa to drive TNF-α expression through PAR-2-dependent ERK1/2 signaling in macrophages and fibroblasts [29,30,31], and extend this paradigm to BMSC-derived chondrocytes. This validation step was performed for every experimental batch; only cultures demonstrating significant TNF-α induction were carried forward for subsequent PalCar intervention experiments.

### 3.4. PalCar Suppresses FXa-Induced PAR-2 Expression

**Figure 2:**
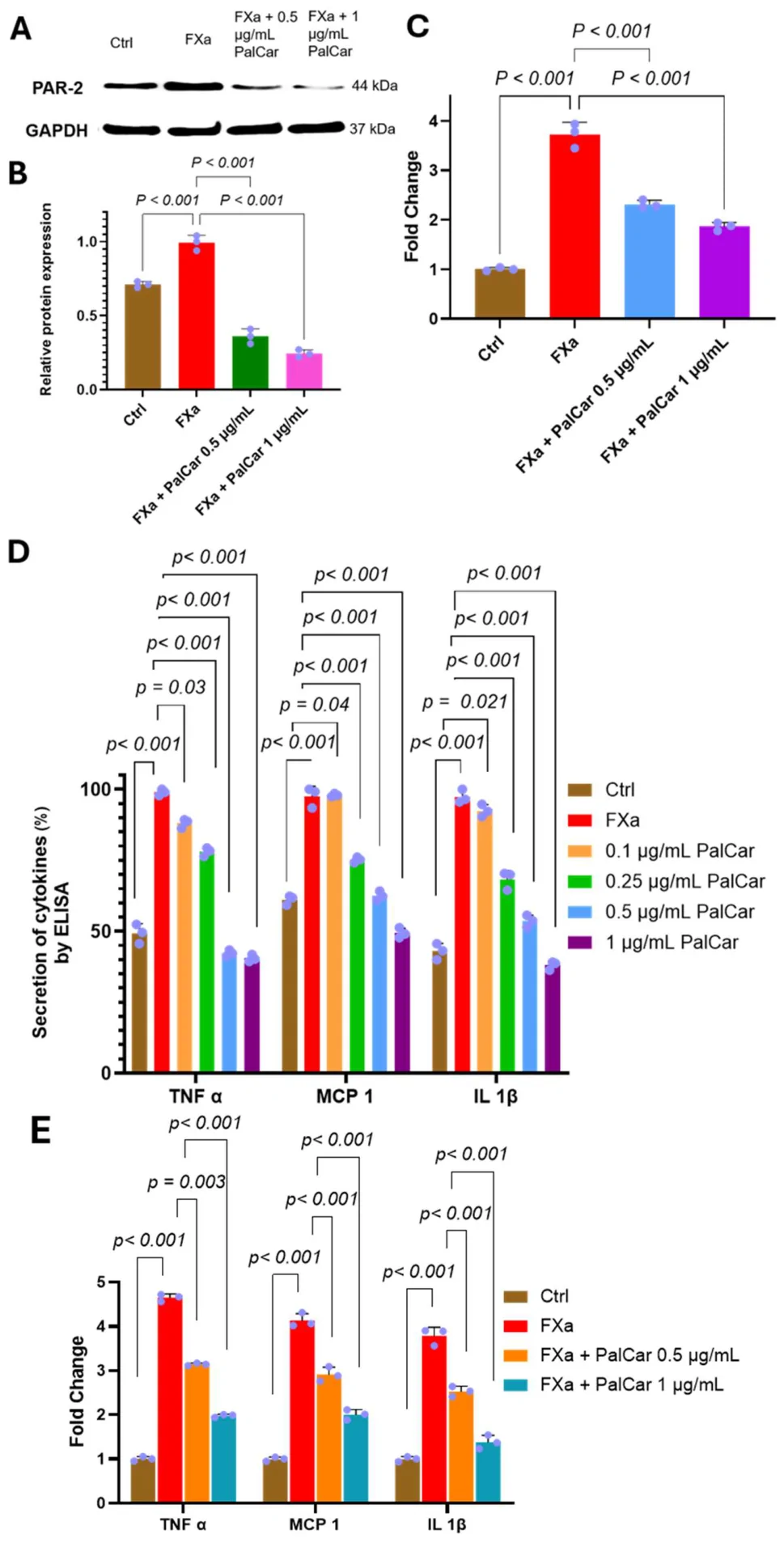
PalCar suppresses FXa-induced PAR-2 expression and cytokine production in BMSC-derived chondrocytes. (**A**) Representative Western blot of PAR-2 (44 kDa) expression in untreated chondrocytes (Ctrl), FXa-treated cells (1 U/mL, 24 h; FXa), and cells co-treated with FXa plus PalCar at 0.5 µg/mL or 1.0 µg/mL, as indicated above each lane. GAPDH (37 kDa) was used as the loading control. (**B**) Densitometric analysis of PAR-2 protein expression, normalised to GAPDH and expressed relative to the untreated control. (**C**) RT-qPCR analysis of PAR-2 mRNA expression, expressed as fold change. (**D**) ELISA quantification of TNF-α, MCP-1 and IL-1β secreted into the conditioned medium of untreated chondrocytes (Ctrl), FXa-stimulated chondrocytes and FXa-stimulated chondrocytes co-treated with PalCar at 0.1, 0.25, 0.5 and 1.0 µg/mL; cytokine levels are expressed as a percentage of the FXa-stimulated control. (**E**) RT-qPCR analysis of TNF-α, MCP-1, and IL-1β mRNA expression, expressed as fold change. Data are presented as mean ± SD from *n* = 3 independent biological experiments, with the individual data points for each replicate shown. Statistical comparisons were made by one-way ANOVA followed by Dunnett’s multiple-comparisons test against the FXa-stimulated reference group, and the exact multiplicity-adjusted *p*-values are shown.

FXa stimulation (1 U/mL, 24 h) markedly increased PAR-2 protein expression in BMSC-derived chondrocytes, as shown by Western blotting and densitometric analysis (Figure 2A,B). PalCar treatment significantly attenuated this response, reducing PAR-2 protein expression by approximately 64% at 0.5 µg/mL and 76% at 1.0 µg/mL relative to FXa alone (Figure 2B). Likewise, RT-qPCR analysis showed that PalCar suppressed FXa-induced PAR-2 mRNA expression by approximately 41% and 49% at 0.5 and 1.0 µg/mL, respectively (Figure 2C). Collectively, these data indicate that PalCar inhibits FXa-induced PAR-2 expression at both transcriptional and protein levels.

### 3.5. PalCar Attenuates FXa-Induced Proinflammatory Cytokine Production

ELISA analysis showed that FXa stimulation markedly increased the secretion of TNF-α, IL-1β, and MCP-1 in chondrocyte-conditioned medium, whereas PalCar treatment reduced all three cytokines in a dose-dependent manner (Figure 2D). At 0.5 µg/mL, PalCar reduced TNF-α, MCP-1, and IL-1β levels to 42.2% ± 0.05, 62.3% ± 0.05, and 53.7% ± 0.04 of FXa-stimulated levels, respectively. At 1.0 µg/mL, a greater inhibitory effect was observed for MCP-1 and IL-1β, with levels reduced to 49.02% ± 0.04 and 38.0% ± 0.03, respectively, while TNF-α remained comparably suppressed at 41.8% ± 0.07 (Figure 2D). Consistent with these findings, RT-qPCR analysis demonstrated that FXa significantly increased TNF-α, MCP-1, and IL-1β mRNA expression by approximately 4.6-, 4.0-, and 3.9-fold, respectively, compared with untreated controls, whereas co-treatment with PalCar at 0.5 and 1.0 µg/mL significantly attenuated these inductions (Figure 2E). Collectively, these results indicate that PalCar suppresses FXa-induced inflammatory cytokine production at both the transcript and secreted protein levels.

### 3.6. PalCar Suppresses FXa-Induced ERK Signaling in BMSC-Derived Chondrocytes

**Figure 3:**
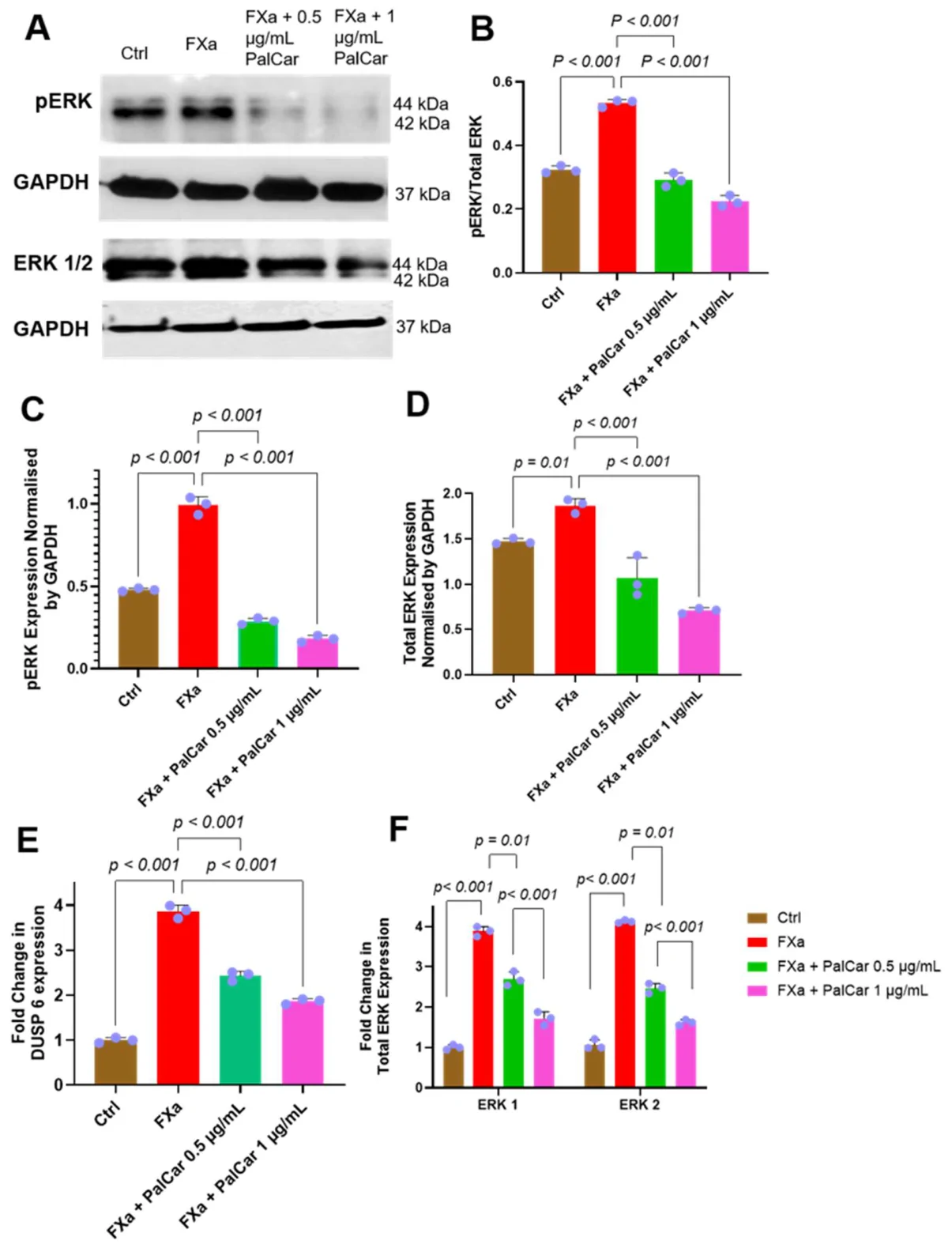
PalCar suppresses FXa-induced ERK signaling in BMSC-derived chondrocytes. (**A**) Representative Western blots showing phospho-ERK (pERK; 44 and 42 kDa), total ERK1/2 (44 and 42 kDa) and GAPDH (37 kDa) in untreated chondrocytes (Ctrl), FXa-stimulated chondrocytes (1 U/mL, 24 h; FXa), and cells co-treated with FXa plus PalCar at 0.5 µg/mL or 1.0 µg/mL, as indicated above each lane. (**B**) Densitometric analysis of the pERK/total ERK ratio. (**C**,**D**) Densitometric quantification of pERK and total ERK1/2 protein expression, respectively, each normalised to GAPDH. (**E**) RT-qPCR analysis of DUSP6 mRNA expression. (**F**) RT-qPCR analysis of ERK1 and ERK2 mRNA expression. Data are presented as mean ± SD from *n* = 3 independent biological experiments, with the individual data points for each replicate shown. Statistical comparisons were made by one-way ANOVA followed by Dunnett’s multiple-comparisons test against the FXa reference group, and the exact multiplicity-adjusted *p*-values are shown.

Because ERK1/2 is a principal downstream effector of PAR-2 signaling, and a key mediator linking receptor activation to proinflammatory gene expression, we next examined whether PalCar modulates the ERK arm of the PAR-2 pathway. Western blot analysis showed that FXa stimulation increased pERK and total ERK1/2 protein expression relative to unstimulated chondrocytes (Figure 3A). Densitometric analysis demonstrated that the pERK/total ERK ratio was significantly elevated by FXa and was reduced by PalCar at both 0.5 and 1.0 µg/mL, with the greater suppression observed at 1.0 µg/mL (Figure 3B). Similarly, pERK protein expression was markedly increased by FXa, and significantly attenuated by PalCar at both concentrations (Figure 3C), while total ERK1/2 protein expression was likewise increased by FXa and reduced by PalCar co-treatment (Figure 3D).

To further substantiate modulation of the ERK pathway at the transcriptional level, we also examined DUSP6, an ERK-responsive dual-specificity phosphatase that serves as a canonical feedback regulator and surrogate readout of ERK pathway activation. FXa markedly increased DUSP6 mRNA expression, whereas PalCar significantly suppressed this induction at both 0.5 and 1.0 µg/mL (Figure 3E). Consistent with these findings, RT-qPCR analysis showed that FXa upregulated ERK1 and ERK2 transcripts by approximately 3.9- and 4.1-fold, respectively, relative to control, and that PalCar significantly attenuated both responses in a concentration-dependent manner (Figure 3F). Collectively, these data indicate that, following its suppressive effects on PAR-2 expression and downstream cytokine production, PalCar also dampens the ERK-centered signaling axis activated by FXa, supporting inhibition of the PAR-2/MAPK inflammatory cascade in chondrocytes.

### 3.7. PalCar Suppresses FXa-Induced SOX4 and ADAMTS5 Expression

**Figure 4:**
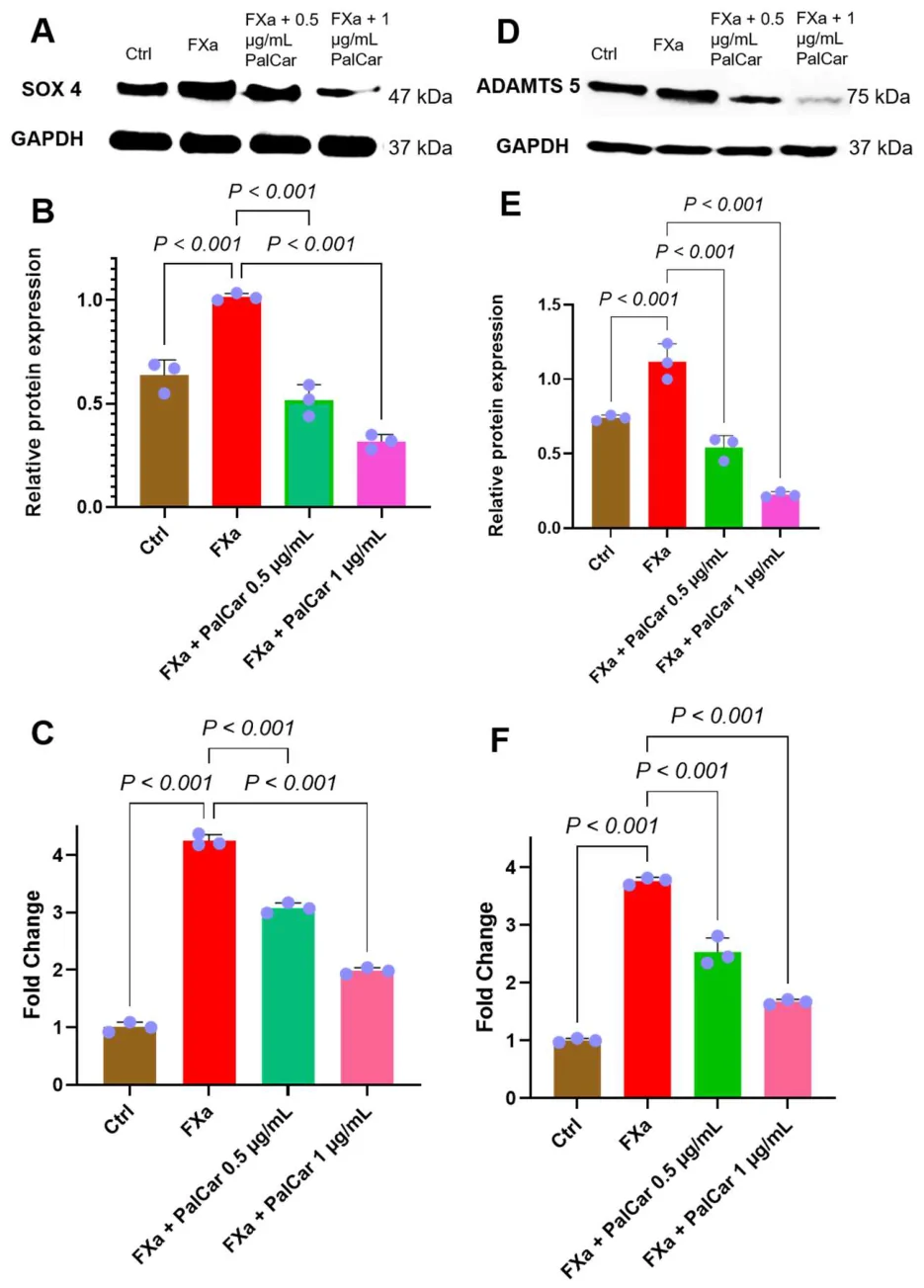
PalCar suppresses FXa-induced SOX4 and ADAMTS5 expression in BMSC-derived chondrocytes. (**A**,**D**) Representative Western blots of SOX4 (47 kDa) and ADAMTS5 (75 kDa) expression in untreated chondrocytes (Ctrl), FXa-treated cells (1 U/mL, 24 h; FXa), and cells co-treated with FXa plus PalCar at 0.5 µg/mL or 1.0 µg/mL, as indicated above each lane. GAPDH (37 kDa) was used as the loading control. (**B**,**E**) Densitometric analysis of SOX4 and ADAMTS5 protein expression, respectively, each normalised to GAPDH. (**C**,**F**) RT-qPCR analysis of SOX4 and ADAMTS5 mRNA expression, respectively, expressed as fold change. Data are presented as mean ± SD from *n* = 3 independent biological experiments, with the individual data points for each replicate shown. Statistical comparisons were made by one-way ANOVA followed by Dunnett’s multiple-comparisons test against the FXa-stimulated reference group, and the exact multiplicity-adjusted *p*-values are shown.

Because inflammatory signaling in chondrocytes ultimately converges on catabolic transcriptional programs that drive matrix degradation, we next examined SOX4 and ADAMTS5 as downstream disease-relevant effectors. SOX4 was selected because it functions as a stress-responsive transcription factor implicated in cartilage catabolism, whereas ADAMTS5 is a principal aggrecanase responsible for extracellular matrix breakdown in OA. Western blot analysis showed that FXa stimulation increased SOX4 protein expression in BMSC-derived chondrocytes, and this induction was significantly attenuated by PalCar at both 0.5 and 1.0 µg/mL (Figure 4A,B). A similar pattern was observed for ADAMTS5 protein expression, which was elevated by FXa and progressively reduced by PalCar co-treatment (Figure 4D,E). RT-qPCR analysis corroborated these findings at the transcript level; FXa increased SOX4 and ADAMTS5 mRNA expression by approximately 4.2- and 3.8-fold, respectively, relative to control, whereas PalCar significantly suppressed both responses at 0.5 and 1.0 µg/mL (Figure 4C,F). Collectively, these data indicate that the anti-inflammatory effects of PalCar extend beyond proximal PAR-2/ERK signaling to downstream catabolic mediators linked to cartilage matrix degradation.

### 3.8. PalCar Suppresses FXa-Induced Surface Expression of RANK and RANKL in Chondrocytes

**Figure 5:**
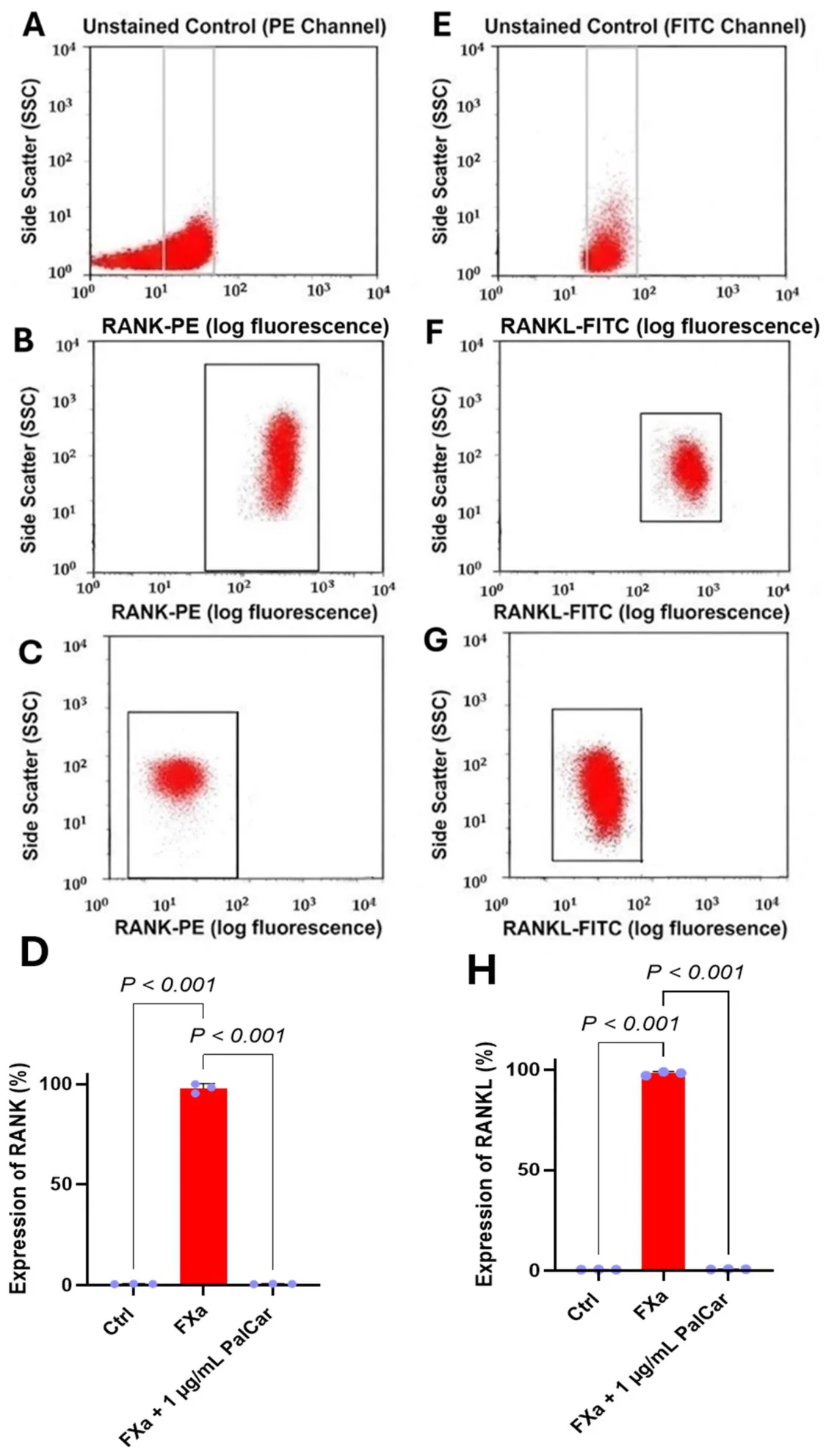
PalCar suppresses FXa-induced surface expression of RANK and RANKL in chondrocytes. (**A**) Unstained control (PE channel) used to set the RANK-positive gate. (**B**,**C**) Representative flow cytometry plots of surface RANK-PE fluorescence in FXa-treated cells (1 U/mL, 24 h) and in cells co-treated with FXa plus PalCar (1.0 µg/mL), respectively. (**D**) Quantification of surface RANK expression, given as the percentage of RANK-positive cells, in untreated chondrocytes (Ctrl), FXa-treated cells (1 U/mL, 24 h) and cells co-treated with FXa plus PalCar (1.0 µg/mL). (**E**) Unstained control (FITC channel) used to set the RANKL-positive gate. (**F**,**G**) Representative flow cytometry plots of surface RANKL-FITC fluorescence in FXa-treated cells (1 U/mL, 24 h) and in cells co-treated with FXa plus PalCar (1.0 µg/mL), respectively. (**H**) Quantification of surface RANKL expression, given as the percentage of RANKL-positive cells, in the same three conditions. Data are presented as mean ± SD from *n* = 3 independent biological experiments, with the individual data points for each replicate shown. Statistical comparisons were made by one-way ANOVA followed by Dunnett’s multiple-comparisons test against the FXa-stimulated reference group, and the exact multiplicity-adjusted *p*-values are shown.

Because OA progression involves not only inflammatory and catabolic signaling within chondrocytes but also pathological cross-talk with subchondral bone remodeling pathways, we next examined the surface expression of RANK and RANKL, two key regulators of osteoimmunologic signaling. Flow cytometric analysis showed minimal basal surface staining for both RANK and RANKL in unstimulated chondrocytes (Figure 5A,E). Following FXa stimulation, a marked rightward shift in the gated positive population was observed for both markers, indicating robust induction of surface RANK and RANKL expression (Figure 5B,F). Co-treatment with PalCar (1.0 µg/mL) substantially diminished this FXa-induced shift, returning the proportion of positively stained cells to near-baseline levels (Figure 5C,G). Quantitative analysis confirmed that RANK and RANKL expression, which increased to maximal levels in the FXa-treated group, was almost completely abrogated by PalCar (Figure 5D,H). These findings indicate that PalCar suppresses FXa-driven activation of the RANK/RANKL axis in chondrocytes, supporting an inhibitory effect not only on inflammatory and catabolic signalling but also on osteochondral remodeling pathways relevant to OA pathobiology.

### 3.9. PalCar Preserves Mitochondrial Membrane Potential in FXa-Stimulated Chondrocytes

**Figure 6:**
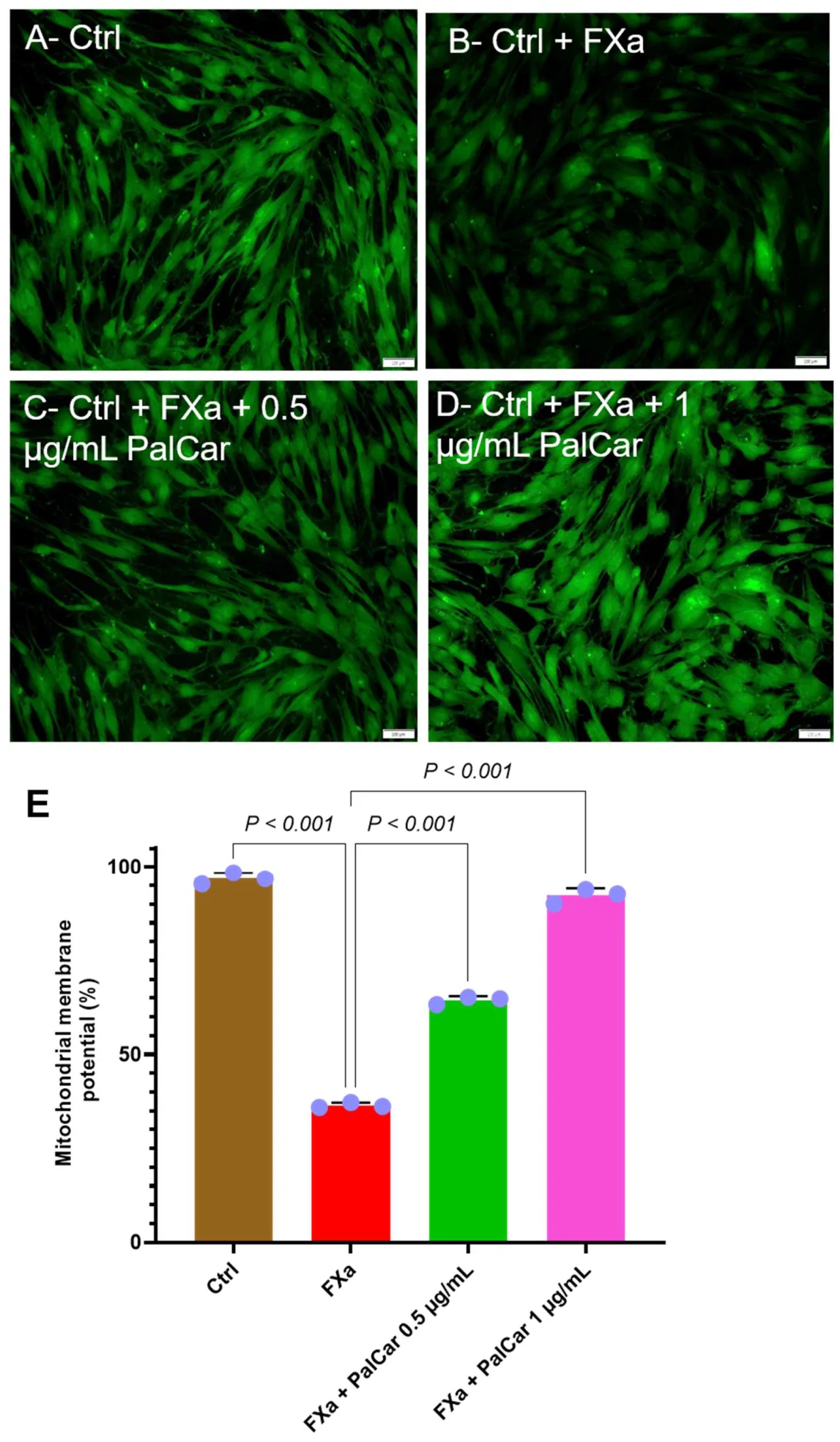
PalCar preserves mitochondrial membrane potential in FXa-stimulated chondrocytes. (**A**–**D**) Representative Rhodamine 123 fluorescence images showing mitochondrial membrane potential in untreated chondrocytes (**A**), FXa-treated cells (1 U/mL, 24 h; (**B**)), and cells co-treated with FXa plus PalCar at 0.5 µg/mL (**C**) or 1.0 µg/mL (**D**). (**E**) Quantification of mitochondrial membrane potential expressed as percentage of control. Data are presented as mean ± SD from *n* = 3 independent biological experiments with the individual data points for each replicate shown. Statistical comparisons were made by one-way ANOVA followed by Dunnett’s multiple-comparisons test against the FXa-stimulated reference group, and the exact multiplicity-adjusted *p*-values are shown. Scale bar 100 µm.

Because PAR-2-driven inflammatory signaling is closely linked to mitochondrial dysfunction and bioenergetic stress, we next examined whether PalCar protects mitochondrial membrane potential (ΔΨm) under FXa stimulation. Rhodamine 123 fluorescence imaging showed strong intracellular fluorescence in untreated chondrocytes, consistent with preserved mitochondrial membrane potential under basal conditions (Figure 6A). In contrast, FXa-treated chondrocytes exhibited a marked reduction in Rhodamine 123 fluorescence intensity, indicating dissipation of ΔΨm and the onset of mitochondrial dysfunction (Figure 6B). Co-treatment with PalCar partially restored fluorescence intensity at 0.5 µg/mL (Figure 6C) and produced a more pronounced recovery at 1.0 µg/mL (Figure 6D), suggesting dose-dependent preservation of mitochondrial integrity. Quantitative analysis confirmed these observations: FXa reduced mitochondrial membrane potential to approximately 35% of control, whereas PalCar restored it to about 64% at 0.5 µg/mL and 93% at 1.0 µg/mL (Figure 6E). These findings indicate that PalCar substantially counteracts FXa-induced mitochondrial depolarization in chondrocytes, supporting the view that inhibition of the FXa–PAR-2 inflammatory axis is accompanied by preservation of mitochondrial function.

### 3.10. PAR-2 Silencing Attenuates, but Does Not Fully Abolish, FXa-Induced Inflammatory Signaling

**Figure 7:**
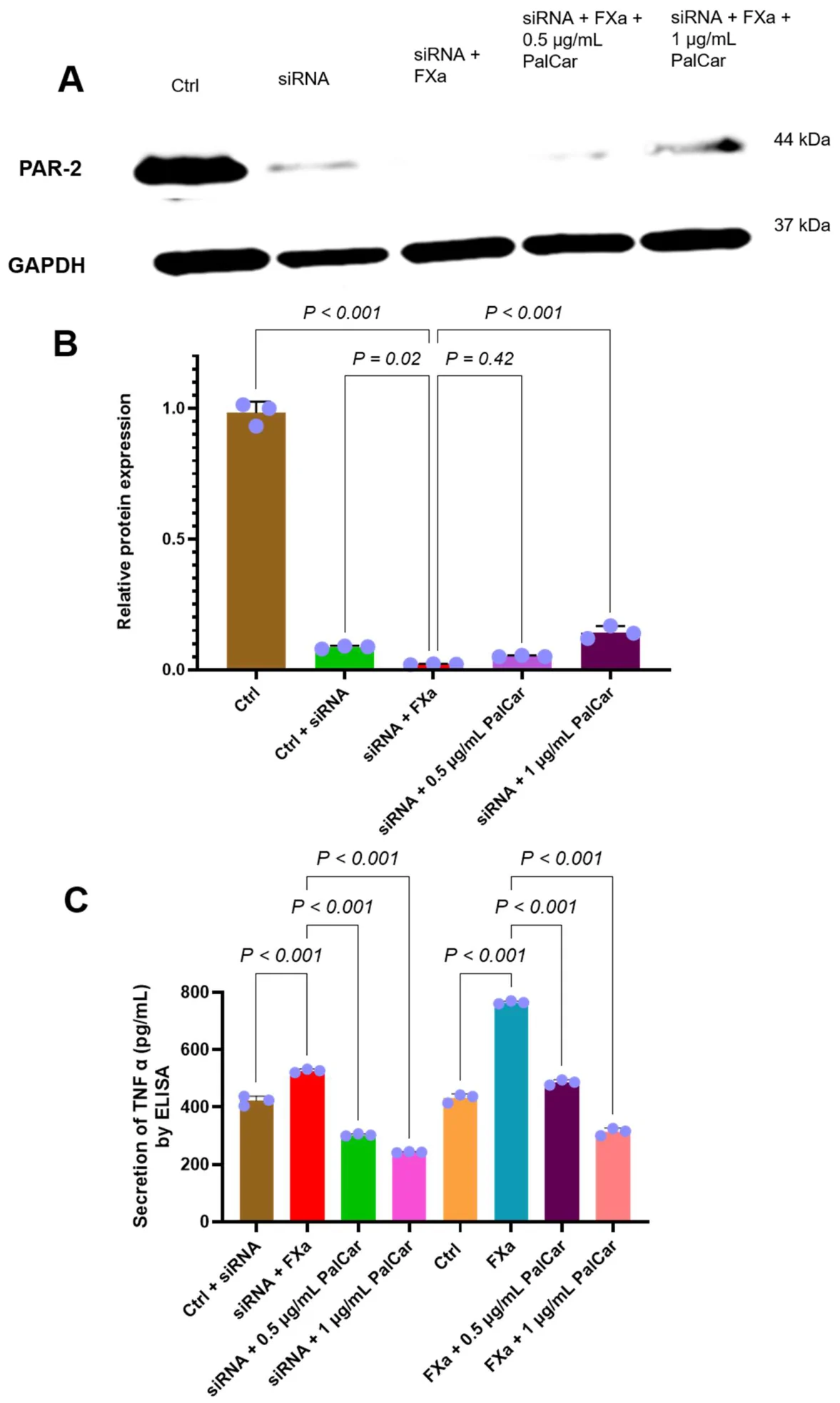
PAR-2 silencing attenuates, but does not abolish, FXa-induced inflammatory signaling in BMSC-derived chondrocytes. (**A**) Representative Western blot showing PAR-2 (44 kDa) protein expression in untreated chondrocytes (Ctrl), chondrocytes transfected with PAR-2 siRNA alone (siRNA), chondrocytes transfected with PAR-2 siRNA and stimulated with FXa (1 U/mL, 24 h; siRNA + FXa), and cells transfected with PAR-2 siRNA and co-treated with FXa plus PalCar at 0.5 µg/mL or 1.0 µg/mL, as indicated above each lane. GAPDH (37 kDa) was used as the loading control. (**B**) Densitometric analysis of PAR-2 protein expression, normalised to GAPDH, across the same five conditions. (**C**) ELISA quantification of TNF-α secretion (pg/mL) in conditioned medium from non-silenced chondrocytes (Ctrl, FXa, FXa + PalCar 0.5 µg/mL and FXa + PalCar 1.0 µg/mL) and from PAR-2-silenced chondrocytes (Ctrl + siRNA, siRNA + FXa, siRNA + PalCar 0.5 µg/mL and siRNA + PalCar 1.0 µg/mL). Data are presented as mean ± SD from *n* = 3 independent biological experiments, with the individual data points for each replicate shown. Statistical comparisons were made by one-way ANOVA followed by Dunnett’s multiple-comparisons test, and the exact multiplicity-adjusted *p*-values, including those for non-significant comparisons, are shown.

To further assess the contribution of PAR-2 to FXa-driven inflammatory responses, chondrocytes were transfected with PAR-2 siRNA and PAR-2 protein expression was examined by Western blotting. siRNA transfection markedly reduced basal PAR-2 expression compared with non-transfected control cells (Figure 7A, lanes 1 vs. 2), confirming effective knockdown. PAR-2 expression remained strongly suppressed following FXa stimulation in siRNA-transfected cells (lane 3), and co-treatment with PalCar at 0.5 or 1.0 µg/mL did not produce any further significant reduction in PAR-2 protein levels beyond that achieved by siRNA alone (lanes 4 and 5; Figure 7A,B).

Functional assessment using TNF-α readout showed that PAR-2 silencing blunted the inflammatory response to FXa, with TNF-α levels reduced in siRNA-transfected cells relative to their corresponding non-silenced groups (Figure 7C). However, FXa continued to elicit a measurable TNF-α response despite PAR-2 knockdown, indicating that PAR-2 is an important, but not exclusive, mediator of FXa-induced inflammatory signaling in chondrocytes. PalCar further reduced TNF-α production in FXa-stimulated cells, although its additional effect in the setting of PAR-2 silencing was comparatively modest. Collectively, these findings support a central role for PAR-2 in FXa-driven chondrocyte inflammation while also suggesting the involvement of additional signaling mechanisms. Importantly, the fact that PalCar produced only a small additional reduction once PAR-2 had already been silenced is mechanistically informative rather than incidental: in a simple epistasis framework, a modulator acting predominantly through a given receptor is expected to lose most of its effect when that receptor is removed. The near-occlusion of PalCar’s action by PAR-2 knockdown therefore provides independent, loss-of-function evidence that PAR-2 is the principal conduit through which PalCar restrains FXa signalling, complementing the gain-of-signal data obtained with FXa challenge alone.

## 4. Discussion

The principal finding of this study is that palmitoylcarnitine (PalCar), an endogenous long-chain acylcarnitine with established FXa-binding properties, attenuates FXa-induced inflammatory and catabolic signaling in BMSC-derived chondrocytes. This suppression operates at multiple levels, including reduction in PAR-2 receptor expression, inhibition of ERK1/2 phosphorylation and transcriptional output, downregulation of catabolic effectors including SOX4 and ADAMTS5, suppression of RANK/RANKL surface expression, and preservation of mitochondrial membrane potential. Taken together, these data identify the FXa–PAR-2–ERK axis as the principal signaling conduit modulated by PalCar in this system, while the siRNA experiments indicate that this pathway accounts for most, but not all, of the FXa-driven inflammatory response.

The biological logic connecting PalCar to FXa signaling modulation is grounded in the prior discovery that long-chain acylcarnitines, including 16:0-acylcarnitine (PalCar), bind directly to FXa at sites outside the Gla domain and inhibit prothrombinase-mediated thrombin generation [23]. That earlier work established PalCar as a physiologically relevant FXa-interacting lipid in the hemostatic context and showed that circulating long-chain acylcarnitines are reduced in venous thrombosis. The present study extends that paradigm by demonstrating that PalCar also modulates FXa-driven signaling through PAR-2 in a non-vascular inflammatory system. Thus, a single endogenous lipid appears capable of attenuating both hemostatic and non-hemostatic FXa functions, underscoring the broader biological significance of acylcarnitine–protease interactions.

PAR-2 occupies a critical position at the interface of coagulation protease biology and inflammatory gene expression. Unlike PAR-1, which is more closely associated with thrombin signaling, PAR-2 is activated by FXa, trypsin, and mast cell tryptase, thereby linking it to a wide range of inflammatory settings including synovial inflammation, airway disease, and gastrointestinal pathology [15,17]. In chondrocytes, PAR-2 activation promotes MAPK signaling, NF-κB-dependent cytokine transcription, and matrix-destructive programs [16]. Our data show that PalCar reduces PAR-2 expression at both mRNA and protein levels following FXa stimulation, thereby attenuating the amplitude of downstream signalling (Figure 2). Whether this effect reflects reduced proteolytic engagement of PAR-2 by FXa, altered receptor trafficking, reduced receptor transcription, or a combination of these mechanisms remains to be defined.

These observations are best read against the wider body of work establishing FXa as a bona fide signalling enzyme rather than a purely haemostatic one. Coagulation proteases generated on cell surfaces act through PARs and tissue factor to alter transcriptional programmes governing growth, migration and inflammation [13,32], and the receptor preference of FXa is context-dependent: in cells expressing PAR-1 but not PAR-2, FXa can drive MAP-kinase phosphorylation and gene induction through PAR-1 [33], whereas in PAR-2-competent systems the FXa–PAR-2 module predominates. Our knockdown data place chondrocytes firmly in the latter category while retaining a measurable PAR-2-independent component, consistent with the recognised signalling promiscuity of FXa. Framing the present findings within this protease-signalling literature clarifies that PalCar does not merely lower a panel of inflammatory readouts but acts at a defined node—the FXa–PAR-2 interface—within a pathway whose biochemistry has been progressively dissected over two decades.

The suppression of ERK1/2 activation is mechanistically consistent with attenuation of upstream PAR-2 signaling, as the Ras–Raf–MEK–ERK cascade is a canonical effector arm of Gαq/βγ-coupled PAR-2 activation [34,35]. PalCar reduced total ERK protein, phospho-ERK levels, and the pERK/total ERK ratio (Figure 3), suggesting inhibition at or upstream of the level of ERK expression and activation. The concordant reduction in ERK1 and ERK2 transcript levels implies transcriptional suppression, consistent with dampened upstream receptor signaling (Figure 3), rather than direct kinase inhibition. These findings position PalCar as a modulator of the MAPK hub through which PAR-2 inflammatory outputs are transduced.

The suppression of SOX4 and ADAMTS5 by PalCar (Figure 4), extends the anti-inflammatory effect of this lipid to catabolic effector pathways directly relevant to cartilage degradation. ADAMTS5 is the principal aggrecanase responsible for proteoglycan cleavage in articular cartilage and is transcriptionally regulated by inflammatory cytokines and MAPK signaling [36]. SOX4, a member of the SRY-related HMG-box transcription factor family, has been implicated in extracellular matrix remodeling and inflammatory transcriptional regulation in joint-associated tissues [37]. The coordinate downregulation of both mediators at protein and mRNA levels indicates that PalCar suppresses a broader inflammatory-catabolic transcriptional program downstream of FXa–PAR-2 signaling.

With respect to the cartilage matrix specifically, the present study already interrogates the principal molecular determinants of extracellular-matrix (ECM) integrity rather than inflammatory readouts alone. ADAMTS5, quantified here at both protein and transcript level, is the dominant aggrecanase driving the aggrecan depletion that constitutes the earliest and rate-limiting event in osteoarthritic cartilage breakdown, so its FXa-induced upregulation and PalCar-dependent suppression represent direct molecular evidence at the level of the matrix-degrading effector itself; in parallel, type II collagen (COL2A1), the principal fibrillar protein of the hyaline matrix, is shown to be catabolically regulated by FXa through the same axis (Supplementary Figure S1), while the sulfated glycosaminoglycan and metachromatic proteoglycan content of the differentiated matrix are documented histologically (Figure 1). These readouts collectively span the aggrecanase, the fibrillar-collagen, and the proteoglycan compartments that the suggested degradation markers are intended to probe, with aggrecanase-mediated aggrecan loss known to precede and sensitise the collagen network to subsequent matrix-metalloproteinase attack, such that ADAMTS5 captures the initiating and decisive step of the cascade. Consistent with this scope, our conclusions are framed at the molecular and transcriptional level, PalCar constrains the FXa–PAR-2–ERK-driven catabolic programme governing these ECM effectors, rather than asserting a directly measured change in net matrix turnover, quantitative assessment of which, through aggrecan and collagen neoepitope release, glycosaminoglycan efflux, and matrix-metalloproteinase activity, constitutes a defined direction for future study.

The reduction in RANK and RANKL surface expression by PalCar (Figure 5), adds an important osteoimmunologic dimension to these findings. While the RANK/RANKL axis is best known for its role in osteoclastogenesis and subchondral bone turnover, it is increasingly recognized as part of the inflammatory signaling milieu associated with OA progression [38]. Our observation that FXa induces, and PalCar suppresses, both RANK and RANKL expression (Figure 5), suggests that FXa–PAR-2 signaling may extend beyond cytokine induction and matrix catabolism to pathways linking cartilage inflammation with osteochondral remodeling. Thus, PalCar may attenuate not only inflammatory and catabolic responses within chondrocytes, but also molecular signals relevant to pathological cartilage–bone cross-talk.

The restoration of mitochondrial membrane potential by PalCar is noteworthy because mitochondrial dysfunction is increasingly recognized as both a consequence and an amplifier of inflammatory signaling in OA chondrocytes [10,39]. Loss of ΔΨm compromises oxidative phosphorylation, enhances oxidative stress, and favours apoptotic and pro-inflammatory signaling, thereby worsening degenerative outcomes. The observation that PalCar preserves ΔΨm under FXa-induced inflammatory stress (Figure 6), indicates that its protective actions extend beyond cytokine suppression to preservation of cellular bioenergetic integrity. In this respect, PalCar appears to dampen not only inflammatory signal propagation but also one of its major metabolic consequences. This inference should nonetheless be interpreted with caution, since mitochondrial status was assessed here with a single membrane-potential probe (Rhodamine 123); the present data therefore support conservation of mitochondrial membrane potential rather than a comprehensive rescue of mitochondrial function. Orthogonal confirmation with JC-1 or TMRE, together with quantification of reactive oxygen species, cellular ATP, and oxygen consumption by extracellular-flux respirometry, would be required to define the extent of mitochondrial protection and is identified as a priority for future study.

The siRNA knockdown experiments provide further mechanistic resolution (Figure 7). PAR-2 silencing substantially reduced, but did not abolish, FXa-induced inflammatory output, indicating that PAR-2 is the dominant, though not exclusive, receptor mediating FXa signaling in this system. The residual inflammatory activity in PAR-2-silenced cells may reflect contributions from alternative receptors, including PAR-1, which can also respond to FXa under selected conditions [40], or from receptor-independent protease effects on extracellular or pericellular substrates [41,42]. This partial dependence on PAR-2 is consistent with the signaling promiscuity of FXa and suggests that future studies should systematically dissect the relative contributions of PAR-1, PAR-2, and non-receptor targets in chondrocyte inflammatory signaling.

Taken together with these knockdown data, the attribution of PalCar’s action to the FXa–PAR-2–ERK axis rests on the convergence of complementary loss-of-function and pathway-mapping evidence already embedded in the present design, rather than on any single readout. The system is defined by its agonist, since FXa, an established proteolytic activator of PAR-2, was the initiating stimulus, so that the receptor-level input was specified a priori rather than inferred. Genetic silencing of PAR-2 constitutes a target-specific loss-of-function that avoids the off-target liabilities inherent to small-molecule receptor antagonists, and it not only attenuated the FXa response but resolved it into PAR-2-dependent and PAR-2-independent components (Figure 7), thereby delineating the axis in question and quantifying its contribution. The ERK node was interrogated directly and at several levels: PalCar lowered phospho-ERK1/2 and the phospho-ERK/total-ERK ratio and suppressed FXa-induced transcription of DUSP6, an ERK1/2-specific, ERK-driven dual-specificity phosphatase whose expression is a faithful transcriptional readout of ERK pathway flux [43], together with ERK1 and ERK2 transcripts; suppression of an ERK-responsive gene is a direct functional index of diminished ERK signalling and renders a dedicated ERK-inhibitor arm confirmatory of a node already measured. Finally, the molecular point of action of PalCar is defined upstream, at the protease itself, since it is an endogenous long-chain acylcarnitine that binds FXa outside the Gla domain and constrains its activity [23], so that its suppression of PAR-2 and ERK is mechanistically coherent with reduced FXa–PAR-2 input rather than with a parallel or off-target route. Consistent with this, the direction and pattern of PalCar’s effects phenocopy genetic PAR-2 ablation across every measured node-receptor expression, ERK activation, catabolic effectors, RANK/RANKL, and mitochondrial membrane potential, a concordance that a mechanism operating outside the axis would not be expected to reproduce.

The pharmacological and mutational tools that might be applied, a PAR-2 agonist or antagonist, a small-molecule FXa inhibitor, catalytically inactive FXa, or an ERK inhibitor, would provide orthogonal triangulation of this conclusion, and each maps onto an element the present data already address: FXa itself is the defining PAR-2 agonist; PAR-2 knockdown supplies receptor-specific loss-of-function; the ERK-responsive DUSP6 readout reports ERK-pathway suppression; and PalCar operates as an endogenous FXa-directed inhibitor whose proteolytic mechanism of PAR-2 activation is established in the cited literature [15,29,30,44]. A catalytically inactive FXa would confirm the well-documented requirement for FXa proteolysis in PAR-2 cleavage, while pharmacological PAR-2 antagonism has independently been shown to be cartilage-protective in osteoarthritis models [18]. The existing combination of a defined protease agonist, receptor-specific genetic ablation, direct ERK-node read-outs, and a biochemically defined upstream target therefore already establishes that PalCar acts through the FXa–PAR-2–ERK axis, and the additional experiments would reinforce rather than alter this assignment. A dedicated biophysical dissection of the PalCar–FXa interaction and its influence on the kinetics of PAR-2 cleavage nonetheless remains a worthwhile objective for future study.

The broader translational context of these findings is also noteworthy. Several epidemiologic and genetic studies suggest that OA is associated with a modestly increased risk of stroke [45,46]. In a population-based cohort, OA was associated with an adjusted hazard ratio of 1.10 for stroke, while in women the adjusted hazard ratio for ischemic stroke reached 1.34. Complementing these observational data, bidirectional Mendelian randomization has provided genetic support for hip OA as a potential risk factor for overall stroke, ischemic stroke, and small-vessel ischemic stroke [45,46]. These associations do not establish a direct PalCar-based mechanism, but they support the concept that OA may intersect broader vascular and thrombo-inflammatory disease biology. In that setting, our demonstration that PalCar suppresses FXa-driven inflammatory signaling in chondrocytes raises the possibility that FXa-sensitive pathways could represent one mechanistic bridge between joint pathology and vascular risk.

This interpretation gains further interest when viewed against the emerging literature on anticoagulant class and OA progression. Boer and colleagues showed that use of vitamin K antagonist anticoagulants was associated with increased OA incidence and progression, with an overall odds ratio of 2.50, including increased risk for both knee OA and hip OA, and concluded that these findings support consideration of direct oral anticoagulants in favour of VKAs when clinically appropriate [47]. Importantly, that study was grounded in disruption of vitamin K-dependent pathways, particularly matrix Gla protein biology, rather than direct modulation of FXa-driven inflammatory signaling within cartilage. In contrast, PalCar appears to act through direct binding to FXa and consequent suppression of FXa-dependent signaling. Conceptually, this distinction is important: an endogenous lipid that attenuates FXa activity without antagonising vitamin K biology may represent a mechanistically different way of targeting coagulation-linked OA pathology.

The previous Blood study is especially relevant here because it established that long-chain acylcarnitines are not merely biomarkers but functional anticoagulant lipids capable of binding and inhibiting FXa [23]. In that study, low plasma acylcarnitine levels were associated with venous thrombosis risk, and mechanistic experiments showed that 16:0-acylcarnitine binds FXa outside the Gla domain and inhibits FXa-driven coagulation. The accompanying commentary further emphasized that these findings pointed to a previously unappreciated pathway in coagulation biology and a potentially informative therapeutic direction [48]. The present work extends that concept from thrombosis biology to OA-relevant inflammation, suggesting that PalCar-sensitive regulation of FXa may have significance beyond classical hemostasis.

At the same time, any translational extrapolation must remain disciplined. Our data do not justify recommending dietary PalCar supplementation as an anticoagulant strategy for patients with OA at the present stage. Rather, the appropriate conclusion is that PalCar emerges as a candidate endogenous modulator of FXa-linked osteo-inflammatory signaling that now warrants testing in more advanced preclinical systems. If future pharmacokinetic, pharmacodynamic, toxicologic, and efficacy studies prove favourable, PalCar or PalCar-inspired molecules could be explored in translational and ultimately clinical models, particularly in settings where OA coexists with elevated thrombo-inflammatory or ischemic risk. Thus, supplementation or therapeutic development should be viewed as a future investigational possibility requiring formal validation, not as a present clinical recommendation.

The translational implications of these findings for osteoarthritis, cerebrovascular risk, and thrombo-inflammatory disease should, however, be framed with corresponding caution. The present data derive from a single reductionist system, an FXa-challenged, BMSC-derived chondrocyte monoculture, and therefore identify a mechanism rather than establish a disease-modifying effect; the associations linking OA to vascular and thrombo-inflammatory pathology remain observational, and any inference that palmitoylcarnitine bridges these processes is, at this stage, a hypothesis to be tested rather than a conclusion to be drawn. What the data do establish is, nonetheless, conceptually novel: to our knowledge, this is the first demonstration that an endogenous, physiologically circulating lipid, a long-chain acylcarnitine hitherto characterised as an anticoagulant, constrains coagulation-protease-driven PAR-2-ERK inflammatory signalling in chondrocytes. This positions palmitoylcarnitine not as a conventional pharmacological inhibitor but as an endogenous metabolic checkpoint coupling systemic lipid and acylcarnitine homeostasis to localised joint inflammation and, potentially, to the wider thrombo-inflammatory interface, a mechanism distinct from, and complementary to, both the anticoagulant and the matrix Gla protein paradigms discussed above.

Substantiating these implications will require staged validation in vivo and in clinical material, and the mechanism defined here specifies that path with some precision. In vivo, the axis is testable in post-traumatic OA models, surgical destabilisation of the medial meniscus [49], anterior cruciate ligament transection, or intra-articular monosodium iodoacetate, with outcomes assessed by standardised OARSI histopathological scoring [50], micro-computed-tomography and magnetic-resonance cartilage morphometry, and quantification of aggrecan and collagen neoepitopes; causal dependence on the receptor can be isolated in protease-activated-receptor-2-deficient (F2rl1−/−) animals, in which PAR-2 depletion already confers protection against experimental OA [51], and the modulator itself evaluated by intra-articular or systemic administration of palmitoylcarnitine with concurrent synovial-fluid and plasma acylcarnitine pharmacokinetics. In clinical material, the hypothesis is addressable through case–control and prospective cohorts, exemplified by resources such as the Osteoarthritis Initiative, profiling plasma and synovial-fluid long-chain acylcarnitines against radiographic severity (Kellgren-Lawrence grade), synovial FXa and PAR-2 expression, and systemic inflammatory indices; through Mendelian randomisation using genetic instruments for circulating acylcarnitine concentrations to interrogate causality for both incident OA and cerebrovascular endpoints, extending the observational and genetic associations already noted [45,46]; and, ultimately, through interventional studies of carnitine or acylcarnitine modulation with structural and inflammatory endpoints. Only evidence of this kind would license the extrapolation of the present mechanism to human OA and its vascular comorbidities, and the findings reported here should be regarded, accordingly, as mechanistic and hypothesis-generating.

It is also relevant that the broader carnitine family carries an established nutraceutical profile in this indication: a systematic review of randomised controlled trials has reported that oral L-carnitine supplementation reduces pain, stiffness and circulating inflammatory markers and improves WOMAC and VAS scores in knee OA [52,53], providing precedent and biological plausibility for carnitine-based intervention. Palmitoylcarnitine is, however, a distinct long-chain acylcarnitine ester that has not itself been evaluated as a supplement or in OA, and the FXa–PAR-2–ERK mechanism defined here is separate from the antioxidant and metabolic actions attributed to L-carnitine. Establishing whether palmitoylcarnitine can be repurposed for OA would therefore require dedicated trials in a carefully defined population, patients with early-to-moderate radiographic knee OA exhibiting an inflammatory, effusion-synovitis phenotype and, ideally, biochemical evidence of intra-articular coagulation-protease activity, and would have to contend with the characteristic difficulties of osteoarthritis trials, including enrichment for the relevant molecular endotype, the slow rate of structural progression that necessitates prolonged follow-up with large cohorts or sensitive imaging and biomarker endpoints, and the need to distinguish symptomatic relief from genuine structure modification.

A point relevant to interpretation concerns the concentrations employed. The FXa stimulus (1 U/mL) was deliberately supraphysiological and continuously unopposed, imposed to generate a maximal, reproducible proteolytic challenge for mechanistic dissection, and is not an estimate of the free FXa concentration within the joint, where FXa is generated transiently and tightly regulated [25,26]; nonetheless, local tissue-factor-driven coagulation activation and thrombin generation are demonstrable in the osteoarthritic joint [27], rendering FXa-directed signalling pathophysiologically pertinent. PalCar was, by contrast, tested at physiologically grounded, low-micromolar concentrations consistent with circulating long-chain acylcarnitines and with its established FXa-binding anticoagulant activity [23]. Because the inhibitor requirement scales with the magnitude and persistence of the enzymatic input, the efficacy of near-physiological PalCar against this maximal challenge likely represents a conservative estimate of its activity against the transient, regulated FXa flux of the joint, a framing that motivates future pharmacokinetic–pharmacodynamic and cartilage-explant studies at clinically anchored exposures.

Several strengths of this study merit emphasis: (A) the use of an endogenous lipid with prior biochemically validated FXa-binding evidence; (B) a multi-level mechanistic analysis spanning receptor expression, MAPK signaling, catabolic mediators, surface receptor profiling, and mitochondrial function; (C) a differentiation-validated chondrocyte model; and (D) receptor knockdown to probe signaling dependence. Limitations include the use of BMSC-derived chondrocytes rather than native articular chondrocytes, the absence of in vivo validation, the incomplete characterization of alternative FXa signaling routes, and the reliance on an in vitro inflammatory model. Future studies should therefore employ primary articular chondrocytes or cartilage explant systems, assess PalCar efficacy in animal OA models, define its effects on PAR-1 and other candidate receptors, and determine whether its observed actions can be translated into joint-protective benefit in vivo.

**Figure 8:**
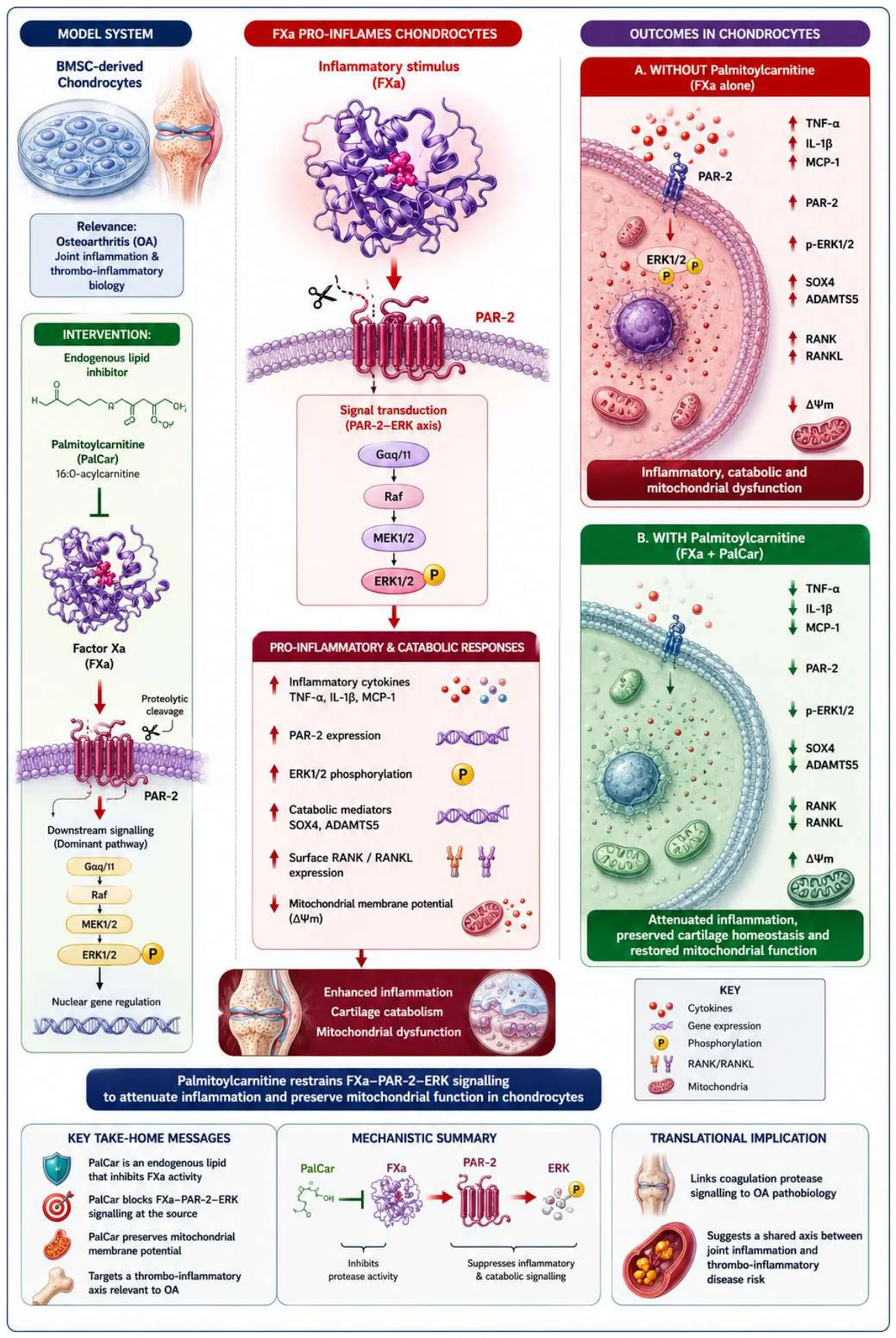
Schematic summary of the principal findings. Factor Xa (FXa) acts as the initiating inflammatory stimulus in BMSC-derived chondrocytes, proteolytically activating protease-activated receptor-2 (PAR-2) and driving the Gαq/11–Raf–MEK1/2–ERK1/2 signalling cascade. This elicits a coordinated pro-inflammatory and catabolic response comprising increased pro-inflammatory cytokines (TNF-α, IL-1β, MCP-1), elevated PAR-2 expression, enhanced ERK1/2 phosphorylation, upregulation of the catabolic mediators SOX4 and ADAMTS5, increased surface RANK and RANKL, and dissipation of the mitochondrial membrane potential (ΔΨm). The endogenous long-chain acylcarnitine palmitoylcarnitine (PalCar; 16:0-acylcarnitine) binds FXa and constrains this FXa–PAR-2–ERK axis at its origin. (**A**) Under FXa alone, chondrocytes display an inflammatory, catabolic and mitochondrially dysfunctional phenotype. (**B**) Co-treatment with PalCar attenuates each of these outputs, lowering cytokine, PAR-2, phospho-ERK1/2, SOX4/ADAMTS5 and RANK/RANKL levels and restoring ΔΨm, consistent with preserved cartilage homeostasis. The mechanism is set within its broader translational context, linking coagulation-protease signalling to osteoarthritis and thrombo-inflammatory disease biology. Created in BioRender. Banerjee, Y. (2026). https://BioRender.com/riqy1gk (accessed on 23 July 2026).

The principal findings of this study are summarised schematically in Figure 8. In brief, FXa serves as the initiating inflammatory stimulus that proteolytically engages PAR-2 on BMSC-derived chondrocytes and drives the Gαq/11–Raf–MEK1/2–ERK1/2 cascade, thereby amplifying pro-inflammatory cytokines (TNF-α, IL-1β, MCP-1), the catabolic effectors SOX4 and ADAMTS5, and the RANK/RANKL axis, while dissipating the mitochondrial membrane potential; palmitoylcarnitine, an endogenous FXa-binding long-chain acylcarnitine, constrains this FXa–PAR-2–ERK axis at its origin, attenuating each of these inflammatory and catabolic outputs and preserving mitochondrial membrane potential. The figure contrasts the chondrocyte phenotype under FXa alone with that under FXa plus palmitoylcarnitine and situates the axis within its broader translational context, linking coagulation-protease signalling to osteoarthritic and thrombo-inflammatory disease biology.

In conclusion, palmitoylcarnitine attenuates FXa-induced inflammatory and catabolic signaling in BMSC-derived chondrocytes through suppression of PAR-2 expression, inhibition of ERK1/2 activation, reduction in catabolic effector pathways, suppression of the RANK/RANKL axis, and preservation of mitochondrial membrane potential. Beyond identifying PalCar as a mechanistically coherent endogenous modulator of coagulation protease-driven inflammatory signaling, the present findings also position FXa-sensitive lipid regulation as a plausible intersection between osteoarthritis, thrombosis biology, and vascular risk. Considering the observed association between OA and stroke, the deleterious link between VKA exposure and OA progression, and the established FXa-inhibitory activity of PalCar, further preclinical evaluation of PalCar-responsive pathways in OA and related thrombo-inflammatory states appears justified.

## Data Availability

All data supporting the findings of this study are available within the article and its Supplementary Information. The raw datasets generated and analysed during the current study (uncropped Western blots, densitometry source values, RT-qPCR cycle-threshold data, ELISA readings, flow cytometry files, and fluorescence-intensity measurements) are available from the corresponding author on request.

## Supplementary Materials

The following supporting information can be downloaded at: https://www.mdpi.com/article/10.3390/biomedicines14081680/s1, Table S1. Oligonucleotide primers used for real-time PCR. Figure S1. Validation of COL2A1 (type II collagen) expression in the BMSC-derived chondrocyte model and its responsiveness to factor Xa. Figure S2. Flow cytometry gating strategy for RANK and RANKL surface staining. Figure S3. Uncropped, full-length immunoblots.
