# Supplementary material for: Physiological Lipid Palmitoylcarnitine Constrains Factor Xa Signalling Through PAR-2 to Attenuate ERK-Driven Inflammation and Preserve Mitochondrial Membrane Potential in Chondrocytes
Source: Biomedicines. 2026 Jul 27;14(8):1680. doi: 10.3390/biomedicines14081680 (PMC13510091; doi:10.3390/biomedicines14081680)
Supplement: Supplementary file 1 [file biomedicines-14-01680-s001.zip › biomedicines-4412232-supplementary.pdf]

# Supplementary Information file

**Supplementary Information for**

**Physiological lipid palmitoylcarnitine constrains factor Xa signalling through PAR-2 to attenuate ERK-driven inflammation and preserve mitochondrial membrane potential in chondrocytes**

Rajashree Patnaik, and Yajnavalka Banerjee

## Contents

**Supplementary Table S1.** Oligonucleotide primers used for real-time PCR.

**Supplementary Figure S1.** Validation of COL2A1 (type II collagen) expression in the BMSC-derived chondrocyte model and its responsiveness to factor Xa.

**Supplementary Figure S2.** Flow cytometry gating strategy for RANK and RANKL surface staining.

**Supplementary Figure S3.** Uncropped, full-length immunoblots.

**Table S1. Oligonucleotide primers used for real-time PCR.**

Primer sequences used for amplification of PAR-2, GAPDH, DUSP6, ERK1, ERK2, TNF-α, IL-1β, MCP-1, SOX4, and ADAMTS5.

| Gene | Primer Type | Sequence | Accession | E-value | Bit Score |
|---|---|---|---|---|---|
| **PAR-2** | Forward (5'-3')<br>Reverse (5'-3') | CTCCTCTCTGTCATCTGGTTCC<br>TGCACACTGAGGCAGGTCATGA | NM-005242 | 0.0 | 2861 |
| **GAPDH** | Forward (5'-3')<br>Reverse (5'-3') | GTCTCCTCTGACTTCAACAGCG<br>ACCACCCTGTTGCTGTAGCCAA | NM_002046 | 0.0 | 2374 |
| **DUSP6** | Forward (5'-3')<br>Reverse (5'-3') | GTCCCAAGGCTTTGGAATCTGTC<br>CCTACCAAGACAGGAGTTCTGG | NM_001946 | 0.0 | 3627 |
| **ERK 1** | Forward (5'-3')<br>Reverse (5'-3') | TGGCAAGCACTACCTGGATCAG<br>GCAGAGACTGTAGGTAGTTTCGG | NM_002746 | 0.0 | 1787 |
| **ERK 2** | Forward (5'-3')<br>Reverse (5'-3') | ACACCAACCTCTCGTACATCGG<br>TGGCAGTAGGTCTGGTGCTCAA | NM_002745 | 0.0 | 5881 |
| **TNF α** | Forward (5'-3')<br>Reverse (5'-3') | CTCTTCTGCCTGCTGCACTTTG<br>ATGGGCTACAGGCTTGTCACTC | NM_000594 | 0.0 | 2449 |
| **IL 1β** | Forward (5'-3')<br>Reverse (5'-3') | CCACAGACCTTCCAGGAGAATG<br>GTGCAGTTCAGTGATCGTACAGG | NM_000576 | 0.0 | 2292 |
| **MCP 1** | Forward (5'-3')<br>Reverse (5'-3') | AGAATCACCAGCAGCAAGTGTCC<br>TCCTGAACCCACTTCTGCTTGG | NM_002982 | 0.0 | 1339 |
| **SOX 4** | Forward (5'-3')<br>Reverse (5'-3') | GACATGCACAACGCCGAGATCT<br>GTAGTCAGCCATGTGCTTGAGG | NM_003107 | 0.0 | 4869 |
| **ADAMTS 5** | Forward (5'-3')<br>Reverse (5'-3') | CCTGGTCCAAATGCACTTCAGC<br>TCGTAGGTCTGTCCTGGGAGTT | NM_007038 | 0.0 | 9621 |
| **COL2A1** | Forward (5'-3')<br>Reverse (5'-3') | CCTGGCAAAGATGGTGAGACAG<br>CCTGGTTTTCCACCTTCACCTG | NM_001844 | 0.0 | 5059 |

*PAR-2:* Protease Activated Receptor-2; *GAPDH*: Glyceraldehyde-3-phosphate dehydrogenase; *pERK:* Phosphorylated Extracellular signal-regulated kinase*; ERK 1:* Extracellular signal-regulated kinase 1; *ERK 2:* Extracellular signal-regulated kinase 2; *TNF α:* Tumor Necrosis Factor α; *IL 1β:* Interleukin 1 Beta; *MCP 1:* Monocyte Chemoattractant Protein-1; *SOX 4:* SRY-box transcription factor 4; *ADAMTS 5:* A Disintegrin and Metalloproteinase with Thrombospondin Motifs 5. COL2A1: Collagen type II alpha 1 chain (type II collagen).

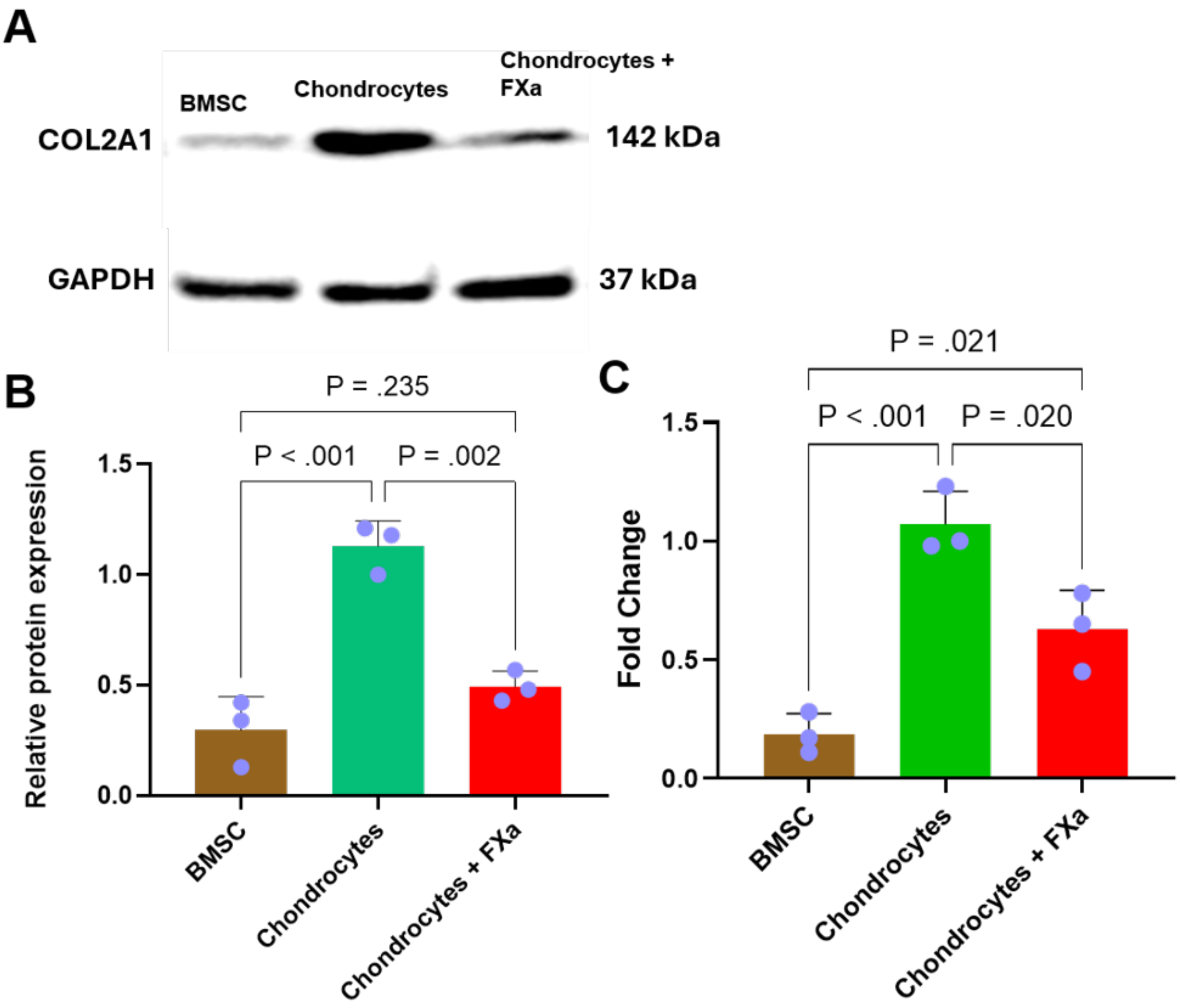


**Supplementary Figure S1.** (A–C) Validation of the BMSC-derived chondrocyte model by COL2A1 (type II collagen) expression and its responsiveness to factor Xa. (A) Representative Western blot of COL2A1 (142 kDa) and GAPDH (37 kDa) in undifferentiated BMSCs, differentiated chondrocytes, and chondrocytes stimulated with FXa (1 U/mL, 24 h), with densitometric quantification of COL2A1 protein expression normalised to GAPDH. (B) RT-qPCR analysis of COL2A1 mRNA expression (2−ΔΔCt method; GAPDH as reference gene) across the same three conditions. Chondrogenic differentiation markedly increased COL2A1 at both protein and transcript levels relative to undifferentiated BMSCs, confirming acquisition of the hyaline chondrocyte phenotype, whereas FXa stimulation significantly reduced COL2A1, consistent with a catabolic effect on cartilage-specific matrix synthesis. Data are presented as mean ± SD from n = 3 independent biological replicates, with the individual data points for each replicate shown. Statistical comparisons were made by one-way ANOVA followed by Tukey's multiple-comparisons test, and the exact multiplicity-adjusted p-values are shown.

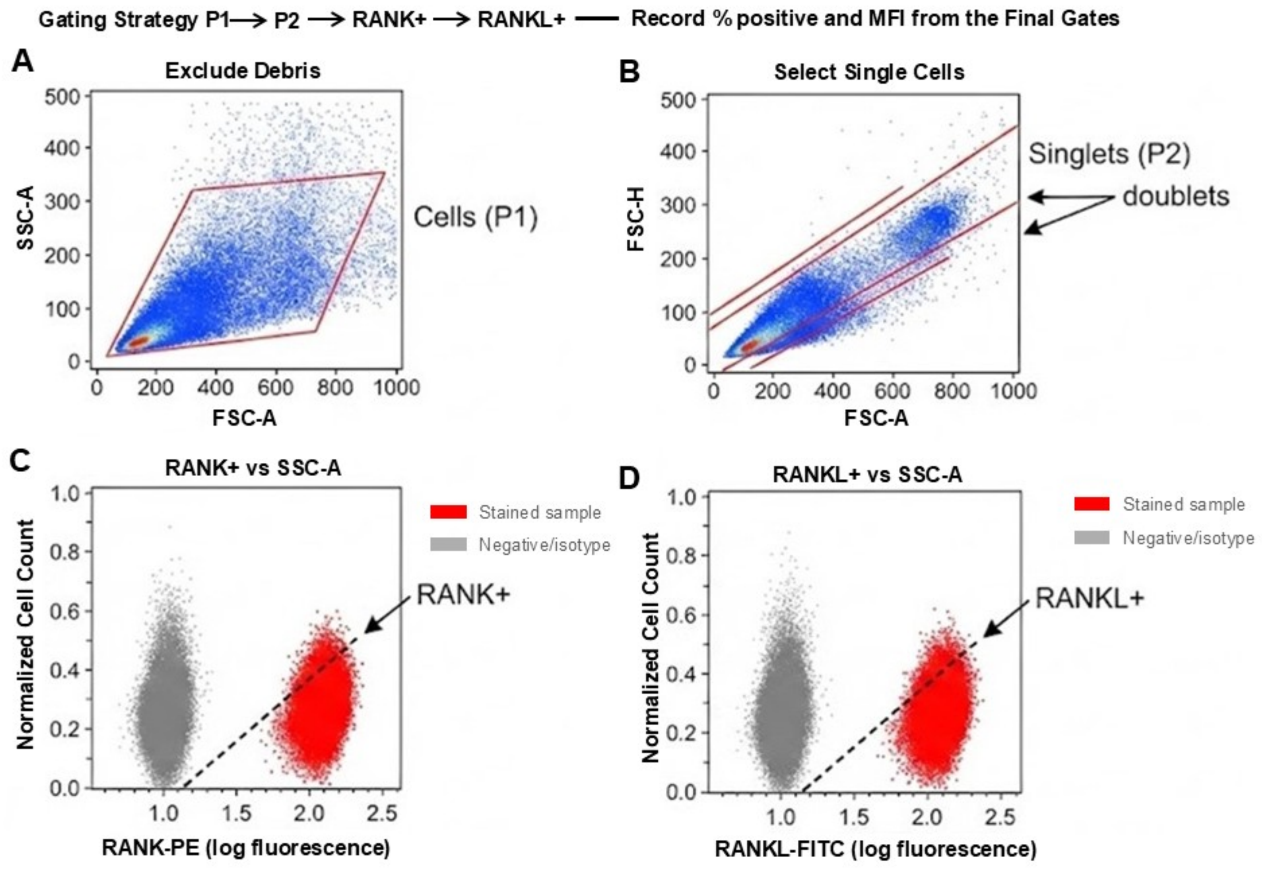


**Supplementary Figure S2. Flow cytometry gating strategy for analysis of FXa-induced RANK and RANKL expression.** Forward scatter–area (FSC-A) versus side scatter–area (SSC-A) dot plots were used to exclude debris and define the main cell population (P1) (A). Singlets were then selected based on FSC-height (FSC-H) versus FSC-A, excluding doublets to generate gate P2 (B). Within P2, RANK-positive cells were identified by plotting normalized cell count versus RANK-PE log fluorescence (C), and RANKL-positive cells by plotting normalized cell count versus RANKL-FITC log fluorescence (D); stained samples are shown in red and negative/isotype controls in grey. Percentages of RANK+ and RANKL+ cells and median fluorescence intensity (MFI) values were recorded from these final gates for quantitative analysis in the main figures.

# Uncropped Blots

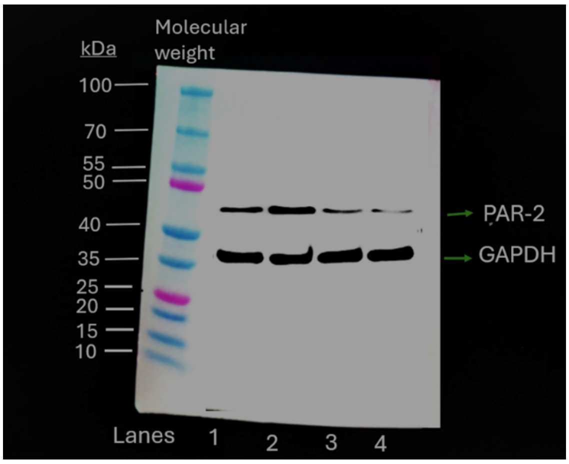


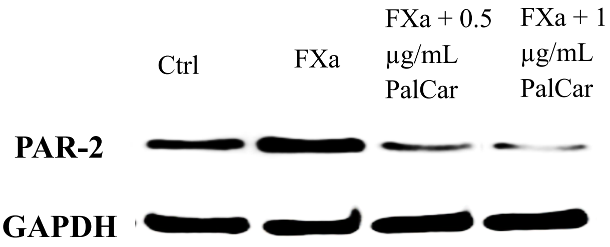


Submitted blot_Figure_2A_PAR-2_lane_map

Uncropped_blot_Figure_2A_PAR-2_lane_map

1- Ctrl
2- FXa
3- FXa + 0.5 µg/mL PalCar
4- FXa + 1 µg/mL PalCar

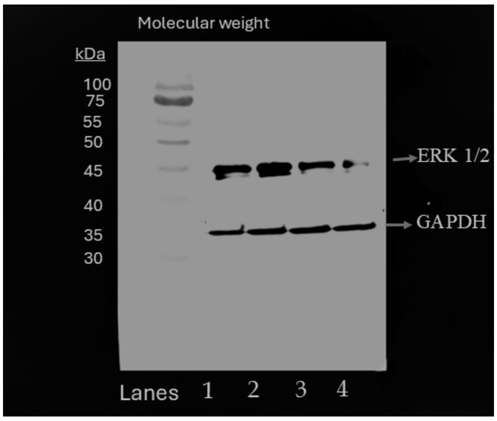


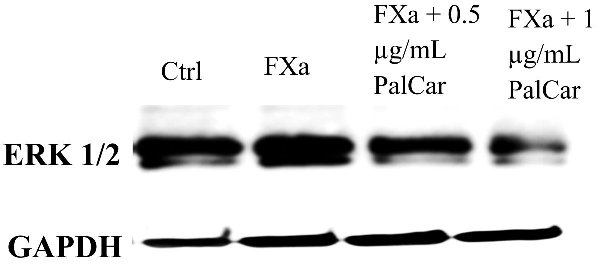


Submitted blot_Figure_3A_ERK_lane_map

Uncropped_blot_Figure_3A_ERK_lane_map

1- Ctrl
2- FXa
3- FXa + 0.5 µg/mL PalCar
4- FXa + 1 µg/mL PalCar

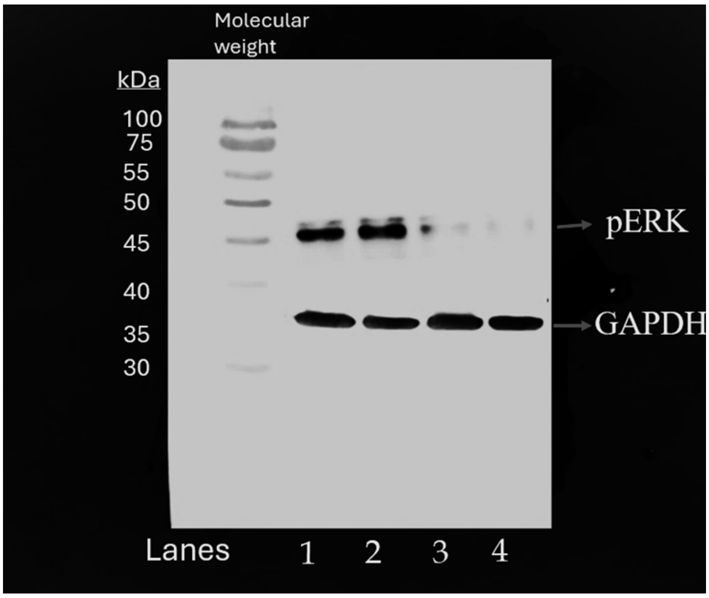


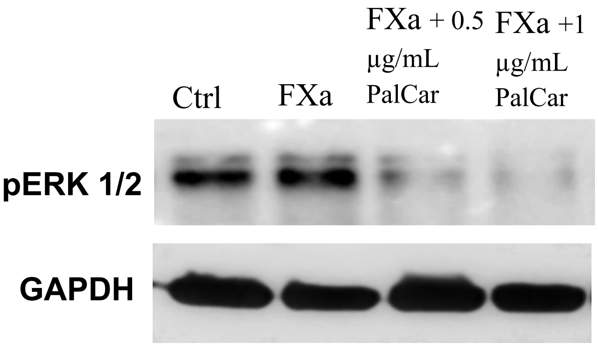


submitted_blot_Figure_3A_pERK_lane_map

Uncropped_blot_Figure_3A_pERK_lane_map

1- Ctrl
2- FXa
3- FXa + 0.5 µg/mL PalCar
4- FXa + 1 µg/mL PalCar

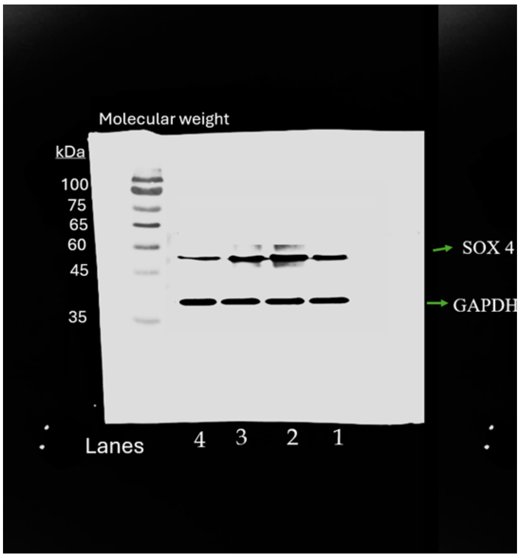


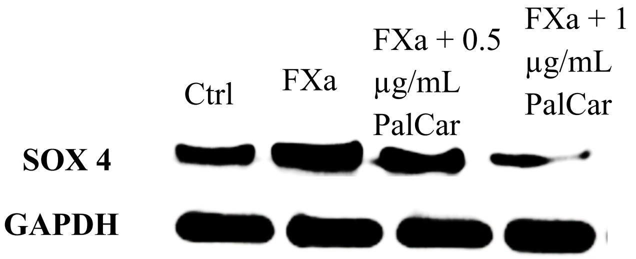


Submitted_blot_Figure_4A_SOX 4_lane_map

Uncropped_blot_Figure_4A_SOX 4_lane_map

1- Ctrl
2- FXa
3- FXa + 0.5 µg/mL PalCar
4- FXa + 1 µg/mL PalCar

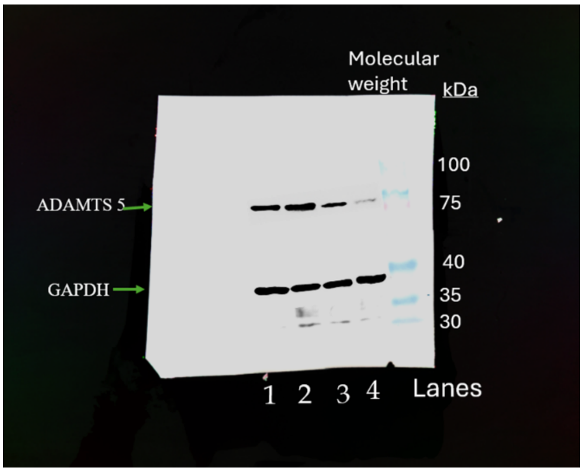


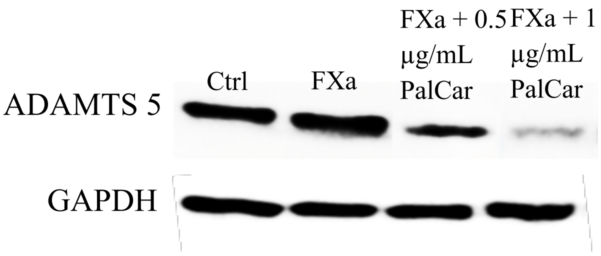


Submitted_blot_Figure_5A_ADAMTS 5_lane_map

Uncropped_blot_Figure_5A_ADAMTS 5_lane_map

1- Ctrl
2- FXa
3- FXa + 0.5 µg/mL PalCar
4- FXa + 1 µg/mL PalCar

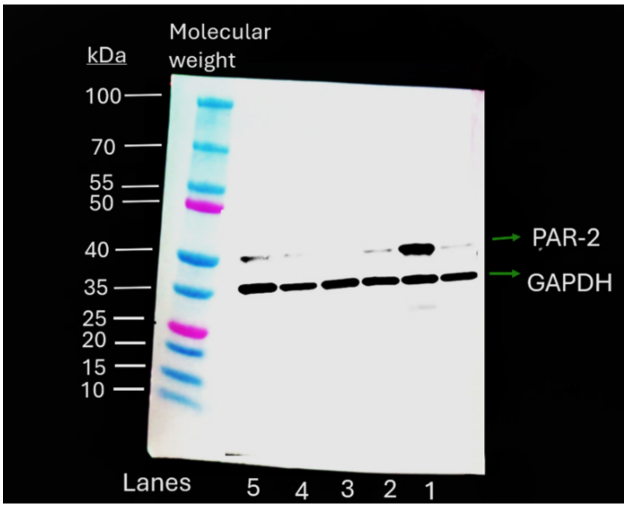


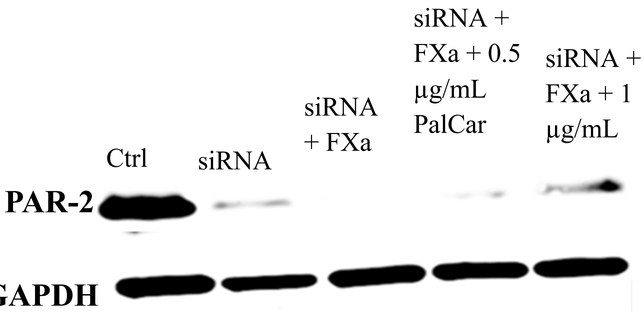


Submitted_blot_Figure_7A_siRNA PAR 2_lane_map

Uncropped_blot_Figure_7A_siRNA PAR 2_lane_map

1- Ctrl
2- siRNA
3- siRNA + FXa
4- FXa + 0.5 µg/mL PalCar
5- FXa + 1 µg/mL PalCar

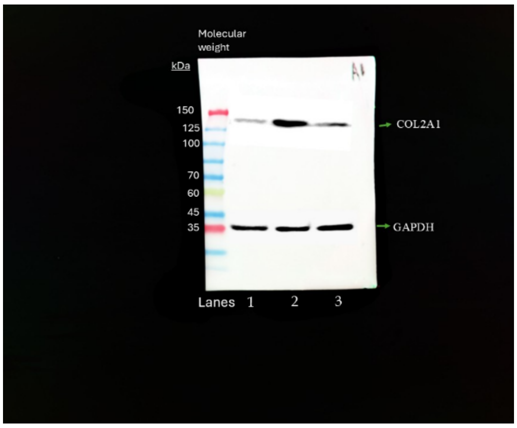


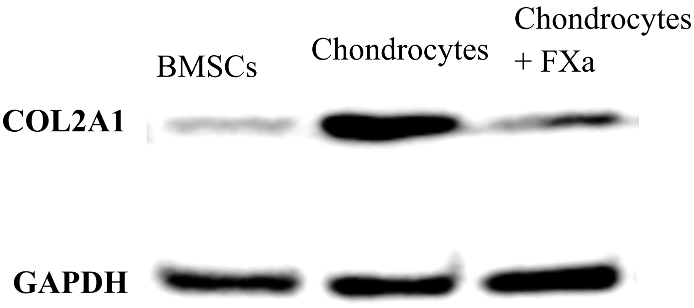


Submitted_blot_Figure_S1_COL2A1_lane_map

Uncropped_blot_Figure_S1_COL2A1_lane_map

1- BMSCs
2- Chondrocytes
3- Chondrocytes + FXa

**Supplementary Figure S3 (Uncropped Blots).** Uncropped, full-length immunoblots. Full membrane images are shown for all immunoblots presented in the main figures, including protease-activated receptor 2 (PAR2), extracellular signal–regulated kinases 1/2 (ERK1/2), phosphorylated ERK1/2 (pERK1/2), SRY-box transcription factor 4 (SOX4), a disintegrin and metalloproteinase with thrombospondin motifs 5 (ADAMTS5), PAR2 siRNA knockdown, and type II collagen (COL2A1). For each target, the complete lane set and corresponding loading control are shown without cropping to demonstrate band specificity and the absence of splicing or selective presentation. Molecular weight markers are included, and lane numbers match those in the associated quantified blots in Figures of the main text. Exposure and contrast settings are identical to those used to generate the analyzed blots, and no additional digital manipulation was applied other than uniform adjustments to brightness/contrast across the entire image.
